# NRF2-dependent gene expression promotes ciliogenesis and Hedgehog signaling

**DOI:** 10.1038/s41598-019-50356-0

**Published:** 2019-09-25

**Authors:** Ana Martin-Hurtado, Raquel Martin-Morales, Natalia Robledinos-Antón, Ruth Blanco, Ines Palacios-Blanco, Isabel Lastres-Becker, Antonio Cuadrado, Francesc R. Garcia-Gonzalo

**Affiliations:** 1 Alberto Sols Biomedical Research Institute UAM-CSIC and Department of Biochemistry, School of Medicine, Autonomous University of Madrid (UAM), Madrid, Spain; 2 La Paz University Hospital Research Institute (IdiPAZ), Madrid, Spain; 3 Centro de Investigación Biomédica en Red sobre Enfermedades Neurodegenerativas (CIBERNED), ISCIII, Madrid, Spain

**Keywords:** Morphogen signalling, Transcriptional regulatory elements

## Abstract

The transcription factor NRF2 is a master regulator of cellular antioxidant and detoxification responses, but it also regulates other processes such as autophagy and pluripotency. In human embryonic stem cells (hESCs), NRF2 antagonizes neuroectoderm differentiation, which only occurs after NRF2 is repressed via a Primary Cilia-Autophagy-NRF2 (PAN) axis. However, the functional connections between NRF2 and primary cilia, microtubule-based plasma membrane protrusions that function as cellular antennae, remain poorly understood. For instance, nothing is known about whether NRF2 affects cilia, or whether cilia regulation of NRF2 extends beyond hESCs. Here, we show that NRF2 and primary cilia reciprocally regulate each other. First, we demonstrate that fibroblasts lacking primary cilia have higher NRF2 activity, which is rescued by autophagy-activating mTOR inhibitors, indicating that the PAN axis also operates in differentiated cells. Furthermore, NRF2 controls cilia formation and function. NRF2-null cells grow fewer and shorter cilia and display impaired Hedgehog signaling, a cilia-dependent pathway. These defects are not due to increased oxidative stress or ciliophagy, but rather to NRF2 promoting expression of multiple ciliogenic and Hedgehog pathway genes. Among these, we focused on GLI2 and GLI3, the transcription factors controlling Hh pathway output. Both their mRNA and protein levels are reduced in NRF2-null cells, consistent with their gene promoters containing consensus ARE sequences predicted to bind NRF2. Moreover, GLI2 and GLI3 fail to accumulate at the ciliary tip of NRF2-null cells upon Hh pathway activation. Given the importance of NRF2 and ciliary signaling in human disease, our data may have important biomedical implications.

## Introduction

Primary cilia are microtubule-based plasma membrane protrusions that function as cell type-specific antennae by accumulating signal receptors and transducers. Structurally, the ciliary membrane is undergirded by the ciliary shaft, or axoneme, consisting of nine microtubule pairs emanating from the basal body, a membrane-anchored centriole^1,2^.

The presence of primary cilia is cell cycle and cell type-dependent^3,4^. In the cell cycle, cilia form during G1/G0 and disassemble before mitosis^3^. Moreover, specific transcription factors (e.g. RFX1-4, FOXJ1) promote ciliogenic gene expression in a cell type-specific manner^4,5^. Ciliogenesis and ciliary maintenance critically depend on a bidirectional trafficking system, known as intraflagellar transport (IFT), which moves cargoes up and down the axoneme^1,2^. To do this, cargoes bind adaptors such as IFT-B and IFT-A complexes, which in turn associate to microtubule motors moving toward (heterotrimeric kinesin-2) or from (cytoplasmic dynein-2) the ciliary tip.

In mammals, the complete absence of cilia is embryonic lethal, as seen in mice lacking essential ciliogenic genes such as *Ift88* and *Kif3a*^6,7^. Less severe congenital ciliary defects lead to ciliopathies, a diverse group of human diseases causing blindness, cystic kidneys, obesity, sterility, polydactyly and brain malformations, among other symptoms^1^. Cilia defects acquired later in life are associated with ailments such as cancer and neurodegeneration^8–11^.

Disruption of Hedgehog (Hh) signaling, a cilia-dependent pathway controlling multiple aspects of embryogenesis and adult stem cell function^7^, explains many (but not all) ciliopathy symptoms and cilia-dependent cancers. Hh ligands, such as Sonic Hedgehog (SHH), act in paracrine fashion by binding to Patched (PTCH1), a ciliary transmembrane protein. Ligand binding prevents PTCH1 from opposing the ciliary accumulation of Smoothened (SMO), a seven transmembrane protein whose activation displaces another G protein-coupled receptor, GPR161, from cilia. This in turn lowers cAMP levels and PKA activity at the basal body, affecting phosphorylation of GLI2 and GLI3, the zinc finger transcription factors mediating Hh pathway output. PKA inactivation also causes GLI2 and GLI3 to accumulate at the ciliary tip, where they dissociate from SUFU, their repressor. From the ciliary tip, GLI2 and GLI3 travel to the ciliary base to meet different fates: GLI2 is processed into a transcriptional activator and translocates to the nucleus to activate target genes, whereas GLI3 is fully degraded in the proteasome, abolishing its basal activity as a transcriptional repressor. Thus, by promoting GLI2 activation and GLI3 non-repression, Hh ligands stimulate expression of target genes, including *Ptch1* and *Gli1*, which provide negative (PTCH1) and positive (GLI1) feedback to the pathway^7^.

Primary cilia also modulate mTOR signaling and autophagy^12–17^. In the kidney, mechanosensory cilia in tubular epithelial cells respond to fluid flow by repressing mTOR, thereby promoting autophagy and reducing cell volume, a homeostatic mechanism perturbed in polycystic kidney disease^13,15,18,19^. Similar pathways occur in other cell types, such as fibroblasts and radial glia^16,17^. During human embryonic stem cell (hESC) differentiation, cell fate choice between mesendoderm and neuroectoderm is also controlled by cilium-dependent autophagy^12^. In the first stages of neuroectoderm progenitor specification, cell cycle lengthening allows cilia to emerge during G1. Cilia in turn activate autophagy, resulting in the inactivation of NRF2, which antagonizes neuroectoderm differentiation. Whether this so-called Primary Cilium-Autophagy-NRF2 (PAN) axis is conserved in other cell types, and whether mTOR is involved in it, has not been addressed^12^.

NRF2 (nuclear factor erythroid 2-related factor 2), encoded by the *Nfe2l2* gene, is a basic region-leucine zipper (bZip) transcription factor best known as a master regulator of cellular antioxidant and detoxification responses^20^. Under normal conditions, NRF2 binding to KEAP1 targets the former for ubiquitin-dependent proteasome degradation. By modifying cysteine residues in KEAP1, oxidative stress, or electrophilic compounds like dimethyl fumarate (DMF), an FDA-approved drug for treatment of multiple sclerosis, disrupt KEAP1-NRF2 binding, leading to NRF2 accumulation^20^. NRF2 then translocates to the nucleus and activates expression of its multiple target genes (hundreds of them) by binding to antioxidant response elements (AREs) in their enhancer regions^20^. Many of these genes encode detoxification enzymes, such as heme oxygenase-1 (*Hmox1*) or the catalytic (*Gclc*) and modulatory (*Gclm*) subunits of glutamate-cysteine ligase, which catalyzes the first step in the biosynthesis of glutathione, a major cellular scavenger of reactive oxygen species (ROS)^20^. NRF2 also targets autophagy genes, which contribute to stress tolerance. Hence, NRF2 is both a regulator of autophagy and an autophagy-regulated protein^12,21,22^.

Likewise, primary cilia both control and are controlled by autophagy^12,14,23,24^. A cilia-specific form of autophagy, termed ciliophagy, plays a key role in lung damage caused by cigarette smoke. HDAC6, a histone deacetylase and ubiquitin-binding autophagy receptor, plays a key role in both ciliophagy and aggrephagy, the autophagic clearance of protein aggregates^23,25,26^. Moreover, autophagy promotes ciliogenesis and lengthens cilia by removing an inhibitory protein, OFD1, from the basal body vicinity^24^.

In view of these reciprocal cilia-autophagy and NRF2-autophagy connections, we wondered whether analogous reciprocal relationships exist between cilia and NRF2. Indeed, we find that fibroblasts lacking cilia have reduced autophagy and increased NRF2 activity, which we rescue with autophagy-activating mTOR inhibitors. Conversely, NRF2-null cells have defects in ciliogenesis and Hh signaling. These defects, rather than being caused by changes in redox stress or ciliophagy, are due to NRF2 promoting expression of multiple ciliogenic and Hh pathway genes.

## Results

### Primary cilia downregulate NRF2 activity via mTOR-dependent autophagy

**Figure 1:**
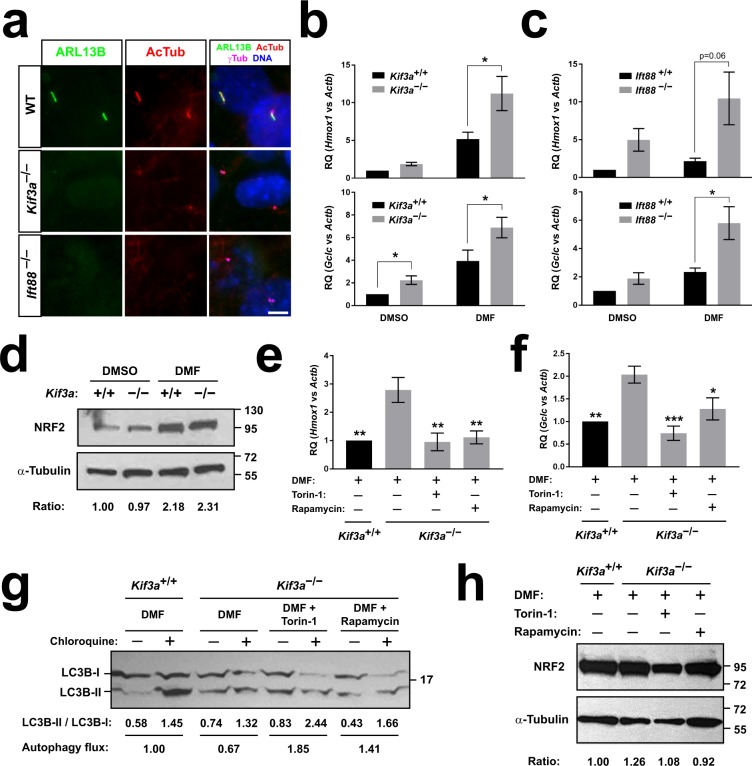
Primary cilia downregulate NRF2 activity via mTOR-dependent autophagy. (**a**) Representative immunofluorescence images of ARL13B, acetylated α-tubulin (AcTub) and γ-tubulin (γTub) in control (*Kif3a*^+/+^, WT), *Kif3a*^−/−^ and *Ift88*^−/−^ MEFs starved 24 h to induce ciliogenesis. Nuclei were counterstained with DAPI. Scale bar, 5μm. (**b**) Expression of NRF2 target genes *Hmox1* and *Gclc* was analyzed by RT-qPCR in *Kif3a*^+/+^ and *Kif3a*^−/−^ MEFs starved for 24 h and then treated for 6 h in starvation medium with vehicle (DMSO) or 20 μM dimethyl fumarate (DMF), as indicated. Data are β-actin (Actb)-normalized relative mRNA levels (mean ± SEM, n = 4 independent experiments). (**c**) Same as in (b) using *Ift88*^+/+^ and *Ift88*^−/−^ MEFs (mean ± SEM, n = 3 independent experiments). (**d**) *Kif3a* MEFs treated as in (b) were analyzed by Western blot with antibodies against NRF2 and α-tubulin, as loading control. Molecular weight markers in kilodaltons are on the right. Quantitation of NRF2/tubulin band intensity ratio is shown below. The compared bands were in the same gel and blot. See methods for details. Uncropped blots are shown in Supplementary Fig. S12. (**e,f**) *Hmox1* (**e**) and *Gclc* (**f**) were analyzed as in (b) in *Kif3a* MEFs treated with DMF (20 μM), Torin-1 (250 nM) or Rapamycin (200 nM), as indicated. Data shown as in (b) (mean ± SEM, n = 6 independent experiments). (**g**) *Kif3a* MEFs were serum-starved for 24 h and then incubated for 4 h in Earle’s balanced salt solution (EBSS) with or without 10 μM Chloroquine and the indicated drugs, as in (e-f). Cell lysates were analyzed by Western blot with anti-LC3B antibodies. Each sample’s LC3B-II/LC3B-I ratio is shown below, together with relative autophagy flux (LC3B ratio increase by Chloroquine, normalized to control). (**h**) *Kif3a* MEFs treated as in (e-f) were analyzed by Western blot and quantitated as in (d). Statistical analysis in (b-c) and (e-f): two-way ANOVA followed by Tukey’s multiple comparisons tests. Asterisks indicate *p < 0.05, **p < 0.01 or ***p < 0.001.

Cells lacking KIF3A, a kinesin-2 subunit, or IFT88, an IFT-B complex component, are completely incapable of growing cilia and are commonly used to study the effects of cilia ablation^1,6,7,27^. We confirmed this using both *Kif3a*^−/−^ and *Ift88*^−/−^ mouse embryonic fibroblasts (MEFs). After 24 hours of starvation, most wild type MEFs displayed primary cilia, in which both axoneme (acetylated α-tubulin, AcTub) and ciliary membrane (ARL13B) markers are seen to emerge from the γ-tubulin-positive basal bodies. In contrast, *Kif3a*^−/−^ and *Ift88*^−/−^ MEFs failed to grow any cilia under the same conditions (Fig. 1a).

To test whether cilia regulate NRF2 activity in MEFs, we measured mRNA levels of heme oxygenase-1 (*Hmox1*) and glutamate-cysteine ligase catalytic subunit (*Gclc*), two well-established transcriptional NRF2 targets^20^, in *Kif3a*^−/−^, *Ift88*^−/−^ and their respective littermate control MEFs (Fig. 1b,c). We tested this in presence or absence of DMF, a known NRF2 activator^20^. Both with and without DMF, cilia-null MEFs displayed consistently higher *Hmox1* and *Gclc* mRNA levels, an effect that was more obvious and significant in presence of DMF (Fig. 1b,c). As expected, DMF raised *Hmox1* and *Gclc*, and it did so with similar fold increases in cilia-null MEFs (Fig. 1b,c). Since these results were observed for both *Kif3a* and *Ift88* MEFs, this suggests that cilia downregulate NRF2 transcriptional activity. Accordingly, another NRF2 target gene, NADPH quinone oxidoreductase-1 (*Nqo1*), was also upregulated, together with *Hmox1*, in both *Kif3a*^−/−^ and *Ift88*^−/−^ MEFs, and this upregulation was seen more clearly in presence of another NRF2 activator, sulforaphane (Supplementary Fig. S1a,b)^20^. Furthermore, *Hmox1* protein levels were also increased in *Ift88*^−/−^ MEFs, and the effect was clearer in sulforaphane-treated cells (Supplementary Fig. S1c).

To see if this increase in NRF2 activity was due to increased NRF2 protein levels, we analyzed the latter by Western blot in *Kif3a*^+/+^ and *Kif3a*^−/−^ MEFs treated or not with DMF. Relative to α-tubulin, NRF2 protein levels were strongly upregulated by DMF in both *Kif3a*^+/+^ and *Kif3a*^−/−^ MEFs, without obvious differences between cell types (Fig. 1d). Hence, the effect of KIF3A on NRF2 activity is not due to changes in NRF2 protein levels.

Since primary cilia downregulation of NRF2 during neuroectoderm differentiation is mediated by autophagy, we next tested whether mTOR inhibitors, which are widely used to activate autophagy, could restore normal NRF2 activity in *Kif3a*^−/−^ MEFs^12,28^. For this, we used both Torin-1 and rapamycin, an ATP-competitive and an allosteric mTOR inhibitor, respectively^29^. Indeed, gene expression of both *Hmox1* and *Gclc* returned to wild type levels upon treatment of *Kif3a*^−/−^ MEFs with either Torin-1 or rapamycin (Fig. 1e,f).

To assess whether changes in autophagic flux may explain the mTOR-dependent effects of cilia on NRF2 activity, we used Western blot to look at the relative levels of LC3B-I and LC3B-II in presence or absence of chloroquine, an autophagolysosome blocker. It is well-established that, during autophagy, LC3B-I is conjugated to phosphatidylethanolamine, thus generating LC3B-II and tethering it to autophagosomes, which evolve into autophagolysosomes where LC3B-II is degraded. Based on this, the chloroquine-induced increase in LC3B-II levels (normalized as LC3B-II/LC3B-I ratio) is a widely accepted measure of autophagic flux. In starved and DMF-treated *Kif3a*^+/+^ MEFs, we saw a clear chloroquine-induced increase in the LC3B-II/LC3B-I ratio, indicating active autophagy (Fig. 1g). Under the same conditions, autophagic flux in *Kif3a*^−/−^ MEFs was reduced by more than 30% relative to the *Kif3a*^+/+^ control. This reduction, however, was rescued by both Torin-1 and rapamycin, in whose presence autophagic flux in *Kif3a*^−/−^ MEFs was even higher than that in *Kif3a*^+/+^ cells (Fig. 1g). Since autophagy flux in these cells shows a strong inverse correlation with NRF2 target expression, these data are consistent with primary cilia downregulating NRF2 by promoting autophagy.

This correlation did not clearly extend to NRF2 protein levels, even though quantitation of NRF2/tubulin ratios showed a slight increase in *Kif3a*^−/−^ relative to *Kif3a*^+/+^ MEFs, an increase that was no longer seen when *Kif3a*^−/−^ MEFs were treated with mTOR inhibitors (Fig. 1h). However, this modest increase seems unlikely to explain the observed increase in NRF2 transcriptional activity.

Altogether, these data suggest that the PAN axis described in differentiating hESCs is also operative in more differentiated cell types, such as MEFs.

### Ciliary repression of NRF2 does not involve Hh signaling

Since primary cilia are required for vertebrate Hh signaling, we also tested whether Hh pathway alterations might be involved in the higher NRF2 activity of *Kif3a*^−/−^ MEFs. In these cells, lack of cilia perturbs proteolytic processing of GLI transcription factors^7^. Since GLI3 is processed into a transcriptional repressor (GLI3R) in a constitutive and cilia-dependent manner, *Kif3a*^−/−^ MEFs fail to form GLI3R, leading to higher unstimulated expression of Hh target genes *Gli1* and *Ptch1* (Supplementary Fig. S2a).

KIF3A and cilia are also required for GLI2 processing, which forms a transcriptional activator (GLI2A) only upon Hh pathway stimulation. Hence, *Kif3a*^−/−^ MEFs fail to form GLI2A and do not induce target gene expression in response to Hh pathway agonists. Nevertheless, Hh target gene expression can be stimulated in these cells by transfecting them with a constitutively active, oncogenic allele of GLI2 known as GLI2ΔN^27^. Indeed, transient expression of GLI2ΔN activated Hh target expression in *Kif3a*^+/+^ and, even more so, in *Kif3a*^−/−^ MEFs, where GLI3R does not counteract its effect (Supplementary Fig. S2a).

However, despite strongly activating Hh signaling in *Kif3a*^+/+^ and *Kif3a*^−/−^ MEFs, GLI2ΔN had no effect on *Hmox1* and *Gclc* gene expression, indicating that NRF2 activity is not affected by GLI2ΔN-induced Hh pathway activation (Supplementary Fig. S2b).

Thus, GLI2 activation does not mediate the effects of cilia on NRF2. Nor does GLI2 inhibition, as GLI2 is transcriptionally inactive in unstimulated MEFs, as confirmed by our observation that GLI2 inhibitor GANT61 does not lower Hh target expression in MEFs not treated with Hh pathway agonists (data not shown)^30^.

Having ruled out GLI2, it was still possible that cilia repress NRF2 activity by promoting GLI3R synthesis. If this were the case, then GLI3R expression in *Kif3a*^−/−^ MEFs should lower NRF2 activity, restoring it to normal. However, this is not the case (Supplementary Fig. S2c,d).

Overall, these data indicate that the negative impact of cilia on NRF2 activity is not related to Hh signaling.

### Ciliogenesis is reduced in NRF2-null cells

**Figure 2:**
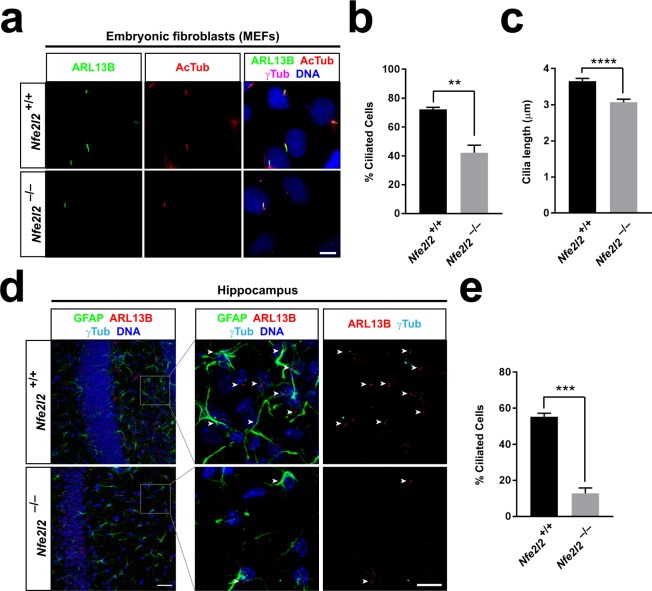
Ciliogenesis is reduced in NRF2-null cells. (**a)** Representative immunofluorescence images for ARL13B, acetylated α-tubulin (AcTub) and γ-tubulin (γTub) in *Nfe2l2*^+/+^ and *Nfe2l2*^−/−^ littermate MEFs starved 24 h to induce ciliogenesis. Nuclei were counterstained with DAPI. Scale bar, 10 μm. (**b**) Quantitation of percentage of ciliated cells from (a) (mean ± SEM of n = 3 independent experiments). (**c**) Quantitation of cilia length from (a) (mean ± SEM of n = 81–85 cilia measured per condition). (**d**) Representative immunofluorescence images for ARL13B, γ-tubulin and astrocyte marker GFAP in hippocampus of 3-month old *Nfe2l2*^+/+^ and *Nfe2l2*^−/−^ mice. Nuclei were counterstained with DAPI. Boxed region in left is magnified on the right. Arrowheads point to cilia. Scale bars, 25 μm (left) and 10 μm (right). (**e**) Quantitation of percentage of ciliated cells from (d) (mean ± SEM, n = 3 mice). Asterisks in (b), (c) and (e): statistical significance in unpaired two-tailed t-tests with **p < 0.01, ***p < 0.001 or ****p < 0.0001.

We next wondered whether NRF2 affects cilia in addition to being affected by them. To test this, we first looked at ciliogenesis in *Nfe2l2*^+/+^ and *Nfe2l2*^−/−^ MEFs. Although *Nfe2l2*^−/−^ MEFs generated cilia that appeared normal by ARL13B, AcTub and γ-tubulin staining (Fig. 2a), the percentage of cells displaying primary cilia was reduced in half relative to *Nfe2l2*^+/+^ cells (Fig. 2b). Moreover, cilia in *Nfe2l2*^−/−^ MEFs were on average 15–20% shorter than control cilia (Fig. 2c). Primary *Nfe2l2*^−/−^ MEFs displayed similar ciliogenic defects, excluding MEF immortalization as their cause (Supplementary Fig. S3).

We then examined whether NRF2 affects ciliogenesis *in vivo*. Since NRF2-null mice are viable and develop normally, we reasoned their embryonic cilia cannot be strongly perturbed^31^. In contrast, adult NRF2-null mice have phenotypes that could potentially be explained by ciliogenic defects. In particular, both NRF2 and cilia play important roles in the hippocampus, affecting cognitive functions^11,32,33^. Thus, we decided to look at hippocampal cilia in coronal sections of three month-old *Nfe2l2*^+/+^ and *Nfe2l2*^−/−^ mouse brains. Moreover, since NRF2 plays a prominent role in astrocytes^34–36^, and ARL13B specifically labels astrocytic cilia in adult mouse brain^37,38^, we focused our study on this cell type, which can be readily identified with the GFAP marker^38^. In both *Nfe2l2*^+/+^ and *Nfe2l2*^−/−^ hippocampus, ARL13B^+^ cilia were indeed only seen in close association with GFAP^+^ astrocytes, confirming the above reports (Fig. 2c)^37,38^. In control sections, about 50% of such GFAP^+^ cells contained a primary cilium, but this percentage was reduced to about 10% in equivalent *Nfe2l2*^−/−^ sections (Fig. 2c,d).

To test whether the ciliogenic effects of NRF2 are cell type-specific, we also looked at the cilia of multiciliated ependymal cells in the brain ventricles of *Nfe2l2*^+/+^ and *Nfe2l2*^−/−^ mice. For this, we stained coronal brain sections for AcTub and basal body marker CEP164. Plentiful cilia lined the ventricular surface in both control and NRF2-null mice, with no apparent differences between the two (Supplementary Fig. S4). This is consistent with the phenotype of NRF2-null mice, which show no signs of hydrocephalus, the usual outcome of ependymal cilia dysfunction^4,31^. Thus, our data suggest that NRF2 is required for optimal ciliogenesis of specific cell types, including astrocytes and fibroblasts.

### Hh signaling is reduced in NRF2-null cells

**Figure 3:**
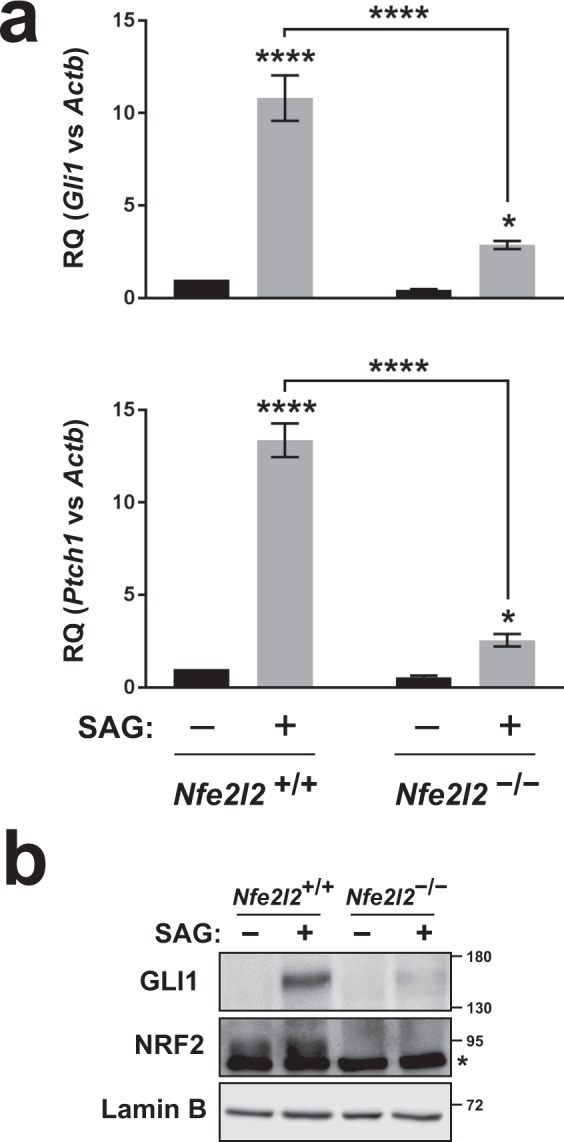
Hh signaling is reduced in NRF2-null cells. (**a)** *Nfe2l2*^+/+^ and *Nfe2l2*^−/−^ MEFs were starved 24 h in presence of DMSO or SAG (200 nM) and expression of Hh target genes *Gli1* (top) and *Ptch1* (bottom) was analyzed by RT-qPCR. Data shown as β-actin (*Actb*)-normalized relative mRNA levels (mean ± SEM, n = 8–9 independent experiments). Statistical analysis: two-way ANOVA followed by Tukey’s multiple comparisons tests. Asterisks: *p < 0.05 or ****p < 0.0001. (**b**) *Nfe2l2* MEFs treated as in (a) were analyzed by Western blot with antibodies for GLI1, NRF2 and Lamin-B as loading control. Molecular weight markers in kilodaltons are shown on the right. Asterisk denotes non-specific band (see methods on NRF2 antibody). Uncropped blots are shown in Supplementary Fig. S12.

Since Hh signaling is cilia-dependent, we next examined Hh pathway responsiveness in *Nfe2l2*^−/−^ MEFs. To do this, we used Smoothened agonist (SAG), a well-established Hh pathway activator^39^. While SAG strongly induced Hh target genes *Gli1* and *Ptch1* in *Nfe2l2*^+/+^ cells, this response was much attenuated in *Nfe2l2*^−/−^ MEFs, even if still significant (Fig. 3a). Very similar results were obtained in primary MEFs, confirming defective Hh responsiveness is not related to MEF immortalization (Supplementary Fig. S5). Moreover, differences in SAG responsiveness were also seen by Western blot of GLI1, whose protein levels were strongly upregulated by SAG in *Nfe2l2*^+/+^ MEFs but much less so in *Nfe2l2*^−/−^ MEFs (Fig. 3b). Thus, NRF2-null cells display defects in both ciliogenesis and Hh signaling.

### ROS, HDAC6, mTOR and PKA inhibitors do not rescue Hh signaling in NRF2-null cells

**Figure 4:**
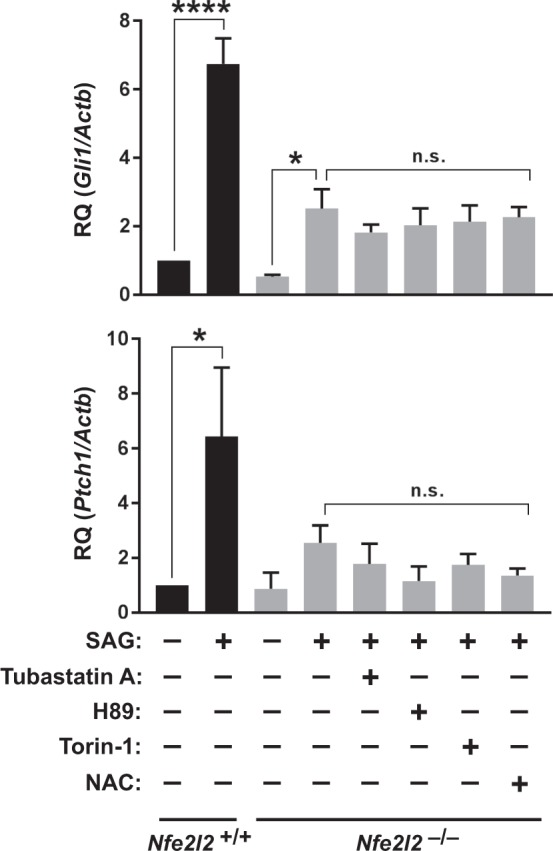
Hh signaling defects in NRF2-null cells are not rescued by HDAC6, PKA, mTOR or ROS inhibitors. *Nfe2l2*^+/+^ and *Nfe2l2*^−/−^ MEFs were starved 24 h in presence of DMSO (vehicle), SAG (200 nM), Tubastatin A (10 μM), H89 (10 μM), Torin-1 (250 nM) or NAC (3 mM), as indicated, and expression of Hh target genes *Gli1* and *Ptch1* was analyzed by RT-qPCR. Data displayed as β-actin-normalized relative mRNA levels (mean ± SEM of n = 5 independent experiments). Statistical analysis: one-way ANOVA followed by Tukey’s multiple comparisons tests. Significance: *p < 0.05, ****p < 0.0001 or not significant (n.s.).

We then asked whether increased oxidative stress in absence of NRF2 was the cause for the sharp decrease in Hh signaling. To address this, we used N-acetyl-cysteine (NAC), a glutathione precursor that promotes ROS scavenging, thereby reducing oxidative stress^40^. NAC treatment of *Nfe2l2*^−/−^ MEFs did not affect their poor SAG responsiveness relative to wild type cells, indicating that the antioxidant functions of NRF2 are not the main reason why it promotes Hh signaling (Fig. 4).

Another possible explanation for the effect of NRF2 on cilia and Hh signaling is that NRF2 affects ciliophagy, which critically depends on HDAC6, a protein that is upregulated in *Nfe2l2*^−/−^ cells and functions as both a protein deacetylase and a ubiquitin-binding autophagy receptor^23,25,26^. If increased HDAC6-dependent ciliophagy was the cause of *Nfe2l2*^−/−^ MEFs displaying ciliogenic and Hh signaling defects, then Tubastatin A, an HDAC6 inhibitor that blocks ciliophagy, should improve Hh responses in these cells^23,41^. However, this is not the case (Fig. 4).

Since PKA, by promoting generation of GLI repressors at the expense of GLI activators, is a negative regulator of Hh signaling, we also tested whether a PKA inhibitor, H89, restored high Hh responsiveness in *Nfe2l2*^−/−^ MEFs^7,42^. The lack of such restoration indicates that NRF2 is required downstream of PKA in the Hh pathway (Fig. 4).

Lastly, given that NRF2 promotes autophagy^21,22^, we tested whether autophagy stimulation with mTOR inhibitor Torin-1 improved Hh signaling in *Nfe2l2*^−/−^ MEFs, but it did not (Fig. 4).

Hence, oxidative stress, HDAC6-dependent ciliophagy, overactive PKA or reduced autophagy do not appear to explain the cilia and Hh phenotypes of *Nfe2l2*^−/−^ MEFs.

### Ciliogenic and Hh pathway gene expression are reduced in NRF2-null cells

**Figure 5:**
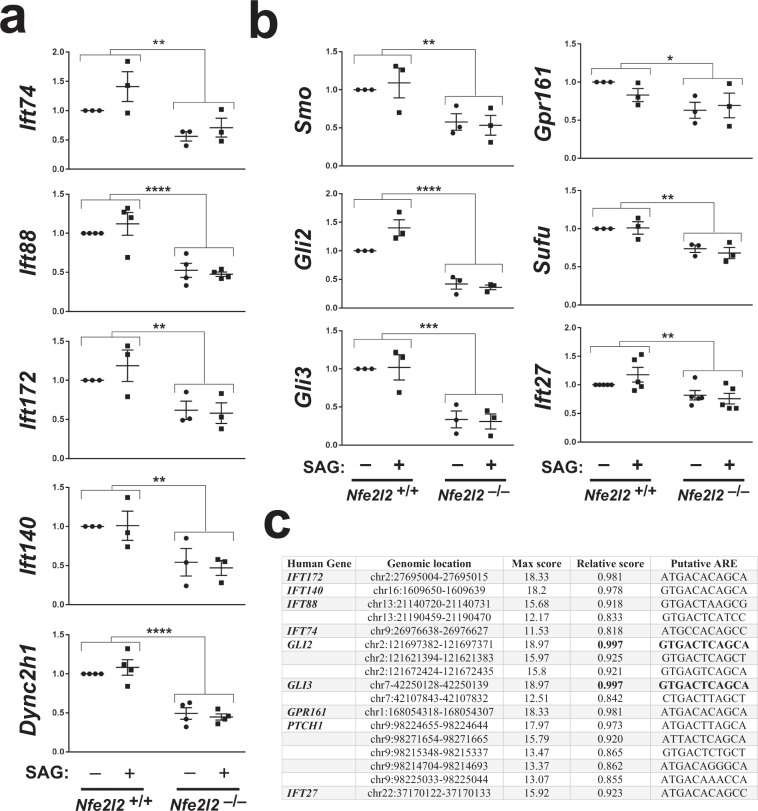
Ciliogenic and Hh pathway gene expression is reduced in NRF2-null cells. (**a)** *Nfe2l2*^+/+^ and *Nfe2l2*^–/–^ MEFs were starved 24 h in presence of DMSO or SAG (200 nM) and expression of the indicated ciliogenic genes was analyzed by RT-qPCR. (**b**) Expression of the indicated Hh pathway genes was analyzed as in (a). All data in (a-b) shown as β-actin-normalized relative mRNA levels (mean ± SEM of n = 3–5 independent experiments). Statistical analysis: two-way ANOVA. Significance between genotypes: *p < 0.05, **p < 0.01, ***p < 0.001 or ****p < 0.0001. SAG treatment did not cause significant differences. (**c**) Putative ARE sequences were identified for the human ciliogenic and Hh pathway genes indicated on the left. Table shows genomic location and sequences of these putative AREs, as well as maximum and relative scores. Only sequences with relative score above 0.8 were considered as hits. No such hits were found for *DYNC2H1*, *SMO*, *SUFU* or *GLI1*. Optimal consensus ARE sequence is (A/G)TGACTCAGCA, according to JASPAR database (see methods for details).

NRF2 is a transcription factor with hundreds of known target genes, so we reasoned that its effects on cilia and Hh signaling might be mediated by changes in gene expression. To test this, we first looked at mRNA levels of ciliogenic genes in *Nfe2l2*^+/+^ and *Nfe2l2*^−/−^ MEFs. These genes encode proteins such as ciliary microtubule motors (*Dync2h1*) and components of intraflagellar transport complexes IFT-B (*Ift74*, *Ift88*, *Ift172*) and IFT-A (*Ift140*). Interestingly, expression of all IFT and motor genes analyzed was significantly lower in *Nfe2l2*^−/−^ relative to *Nfe2l2*^+/+^ MEFs, regardless of whether or not cells were treated with SAG (Fig. 5a). Similar results were obtained in primary MEFs (Supplementary Fig. S6a).

*Nfe2l2*^−/−^ MEFs show a two-fold reduction in ciliogenesis (Fig. 2b), yet their Hh responsiveness is lower by at least 4-fold (Fig. 3a). This suggested that the remaining cilia in *Nfe2l2*^−/−^ MEFs are not fully functional regarding Hh signal transduction. This, we hypothesized, might be due to defects in gene expression of Hh pathway components. To test this, we measured mRNA levels for several Hh pathway mediators, including positive regulators SMO, GLI2 and IFT27, and negative regulators GLI3, SUFU and GPR161^7^. All of them were significantly downregulated in *Nfe2l2*^−/−^ MEFs independently of Hh pathway activation (Fig. 5b). Similar reductions in Hh pathway gene expression were observed in primary MEFs (Supplementary Fig. S6b).

Additionally, we measured gene expression of RFX transcription factors, some of which promote ciliogenesis by binding to X-box motifs in the regulatory regions of many ciliary genes^1,4,5^. Interestingly, two of them were significantly reduced (*Rfx5* and *Rfx7*), one was strongly upregulated (*Rfx8*) and three were unaffected (*Rfx1*, *Rfx2*, *Rfx3*) in *Nfe2l2*^−/−^ MEFs (Supplementary Fig. S7a). For *Rfx4*, absence of NRF2 made no difference in immortalized MEFs, yet it upregulated expression about 100-fold in primary MEFs (Supplementary Fig. S7b). However, as interesting as these changes may be, they seem to bear little or no relationship with our observed ciliogenic and Hh defects, as will be discussed later.

In contrast, the widespread reduction in ciliogenic and Hh pathway gene expression shown in Fig. 5a,b is likely to be a major cause of the ciliogenic and Hh signaling defects of *Nfe2l2*^−/−^ MEFs.

### DMF does not increase basal levels of ciliogenesis and Hh responsiveness

Since NRF2 loss in *Nfe2l2*^−/−^ MEFs causes a reduction in ciliogenesis and Hh signaling, we wondered whether higher NRF2 levels would have the opposite effect, increasing ciliogenesis and Hh pathway output. To test this, we treated *Nfe2l2*^+/+^ MEFs (and *Nfe2l2*^−/−^ MEFs as a negative control) with DMF, which raises NRF2 protein levels (Fig. 1d)^20^. DMF treatment did not significantly increase ciliogenesis in either *Nfe2l2*^+/+^ or *Nfe2l2*^−/−^ MEFs, nor did it affect ciliary length (Supplementary Fig. S8a,b). Likewise, Hh responsiveness was not enhanced by DMF (Supplementary Fig. S8c). Thus, it appears that basal NRF2 levels are not limiting for ciliogenesis or Hh signaling in MEFs, at least under our study conditions.

### DMF stimulates ciliogenic gene expression

Even though DMF did not enhance ciliogenesis or Hh signaling, its upregulation of NRF2 protein levels might still cause an increase in NRF2-dependent gene expression. We therefore tested if DMF affects expression of some of the ciliogenic and Hh pathway genes from Fig. 5a,b. Interestingly, DMF caused a significant increase in *Dync2h1* and *Ift88* mRNA levels in *Nfe2l2*^+/+^ but not *Nfe2l2*^−/−^ MEFs, indicating that the effect depends on NRF2 (Supplementary Fig. S9a). As for Hh pathway genes, we checked mRNA levels of *Smo*, *Gli2* and *Gli3*. Although we did not see any significant DMF-induced increase for any of these genes in control or NRF2-null MEFs, in the former there was a consistent upward trend, which might merit further study (Supplementary Fig. S9b). Altogether, these experiments suggest that NRF2 levels in control cells may be limiting for the expression of some genes but not others.

### Identification of putative AREs in ciliogenic and Hh pathway genes

Given the effects of NRF2 upon ciliogenic and Hh pathway gene expression, we reasoned that some of these genes might be direct targets of NRF2. If so, they should contain ARE sequences in their regulatory regions. To assess this possibility, we analyzed the promoter regions of all ciliogenic and Hh pathway genes whose expression we found reduced in NRF2-null cells. Promoter sequences of the human genes were compared with consensus ARE sequences from the JASPAR database, as previously described^22^.

Remarkably, many of these genes contain putative ARE sequences with high scores relative to the consensus (Fig. 5c). In particular, two genes (*GLI2* and *GLI3*) contain putative AREs with relative scores higher than 0.99, indicating a virtually perfect match with the consensus. Six other genes (*IFT172*, *IFT140*, *IFT88*, *GPR161*, *PTCH1*, and *IFT27*) contain putative AREs with scores above 0.9, whereas *IFT74* has one above 0.8. In contrast, no putative AREs with scores above 0.8 were found in the promoter regions of *DYNC2H1*, *GLI1*, *SUFU* and *SMO*.

Altogether, our bioinformatic analysis supports the idea that some ciliogenic and/or Hh pathway genes are direct NRF2 transcriptional targets.

### GLI2 and GLI3 ciliary localization and protein levels are reduced in NRF2-null cells

**Figure 6:**
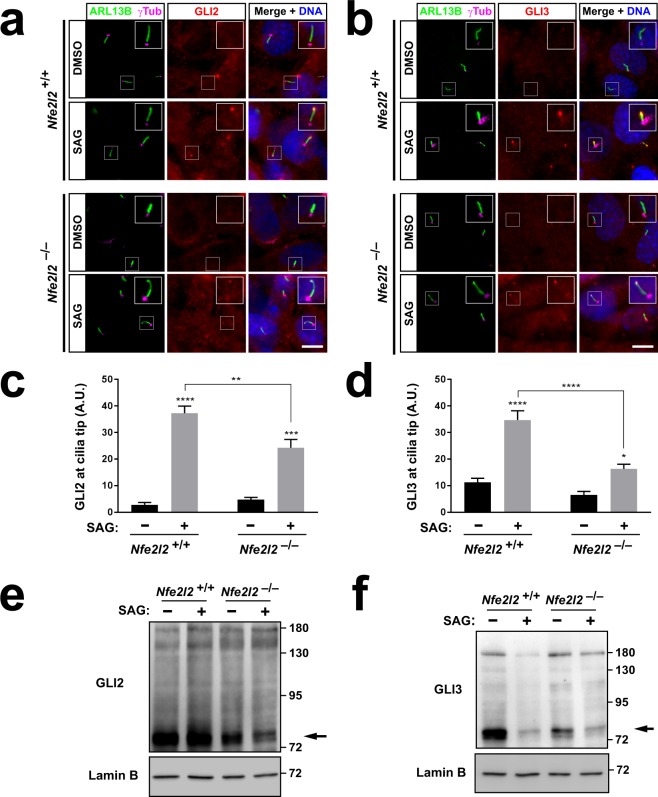
GLI2 and GLI3 ciliary tip accumulation and protein levels are reduced in NRF2-null cells. (**a**,**b)** Representative immunofluorescence images for ARL13B, γ-tubulin and GLI2 (**a**) or GLI3 (**b**) in *Nfe2l2*^+/+^ and *Nfe2l2*^−/−^ littermate MEFs starved 24 h in presence of DMSO (vehicle) or SAG (200 nM). Nuclei were counterstained with DAPI. Top right of each image shows magnification of boxed area. Scale bars, 10 μm. (**c**,**d**) Quantification of GLI2 (**c**) or GLI3 (**d**) signal intensity at ciliary tip from (a,b). Data are mean ± SEM of at least 40 cilia per condition (A.U.: arbitrary units). Statistical analysis: two-way ANOVA followed by Tukey’s multiple comparisons tests. Asterisks: *p < 0.05, **p < 0.01, ***p < 0.001 or ****p < 0.0001. (**e**,**f**) *Nfe2l2*^+/+^ and *Nfe2l2*^−/−^ MEFs were treated as in (a,b) and analyzed by Western blot with GLI2 (**e**) and GLI3 (**f**) antibodies. Most GLI2 and GLI3 are found in their processed forms (arrows), whose levels are reduced in mutant MEFs (and by SAG treatment for GLI3, as expected). Lamin B was used as loading control. Molecular weight markers in kDa are on the right.

Of all the Hh pathway genes whose expression is reduced in NRF2-null cells, GLI2 and GLI3 are potentially the most significant, as they act downstream of all others, directly controlling Hh target gene expression^7^. We therefore looked at GLI2 and GLI3 proteins in *Nfe2l2*^−/−^ MEFs. Both GLI2 and GLI3 are known to accumulate at the ciliary tip upon Hh pathway activation^7,43^. This accumulation is important for GLI2 to be processed into the transcriptionally active GLI2A, and for GLI3 to stop being processed into a repressor, GLI3R, and undergo full proteasomal degradation instead^7,44–46^. Thus, failure of GLI2 and/or GLI3 to accumulate at the ciliary tip might well explain the low Hh responsiveness of *Nfe2l2*^−/−^ MEFs. To test this hypothesis, we performed immunofluorescence for GLI2 and GLI3 in *Nfe2l2* MEFs treated with either vehicle or SAG. In *Nfe2l2*^+/+^ MEFs, SAG induced a strong accumulation of both GLI2 and GLI3 at the ciliary tip. By contrast, their accumulation in SAG-treated *Nfe2l2*^−/−^ MEFs was strongly and significantly reduced (Fig. 6a–d).

Western blot analysis of GLI2 and GLI3 confirmed a strong reduction in the overall basal levels of these proteins, especially of their proteolytically processed lower molecular weight forms (Fig. 6e,f). Moreover, *Nfe2l2*^−/−^ MEFs appeared to react differently to SAG than *Nfe2l2*^+/+^ MEFs. In control cells, as previously shown, SAG did not affect the pattern of GLI2 bands^47^, but caused a marked reduction in GLI3 levels^48^. On the other hand, *Nfe2l2*^−/−^ MEFs responded to SAG by reducing the processed forms of GLI2 while having only a modest effect on GLI3 (Fig. 6e,f). Together with the immunofluorescence data, these results suggest that GLI2 and GLI3 ciliary accumulation, processing and protein levels are abnormal in NRF2-null cells.

Finally, we also carried out immunofluorescence and Western blot with IFT88 and SMO antibodies. Despite the observed lower mRNA levels in *Nfe2l2*^−/−^ MEFs relative to control (Fig. 5a,b), we saw no such differences in either ciliary IFT88 intensity (Supplementary Fig. S10a,b), ciliary SMO intensity with or without SAG (Supplementary Fig. S10c,d), or total levels of these proteins as seen by Western blot in presence or absence of DMF (Supplementary Fig. S10e). This might reflect compensatory mechanisms that maintain protein homeostasis despite altered mRNA levels.

## Discussion

In pluripotent hESCs, cell fate choice between mesendoderm and ectoderm lineages depends on the PAN axis, whereby early emergence of cilia in some cells stimulates autophagy, thereby downregulating NRF2 activity and committing those cells to neuroectoderm^12^. Here, we have shown data suggesting that the PAN axis is also at work in more differentiated cell types, including fibroblasts. In support of this, NRF2 transcriptional activity is higher in cells unable to grow cilia (Fig. 1a–c), whose autophagic flux is reduced (Fig. 1g). Moreover, mTOR inhibitors, which raise autophagic flux in cilia-null cells (Fig. 1g), fully rescue the abnormal NRF2 activity of these cells (Fig. 1e,f).

If the PAN axis is not restricted to hESCs, then what is its biological role beyond early embryogenesis? In stem cells, it may control cell fate choices in a similar way to what it does in hESCs, except that the relevant NRF2 target genes would differ in each case (in hESCs it promotes expression of pluripotency genes OCT4 and NANOG, which are epigenetically silenced in more differentiated cells^12^).

In terminally differentiated cells, the PAN axis might convey information about ciliary integrity or stress. Since genetic deletion of cilia reduces autophagy and raises NRF2 activity, something similar might occur by accidental or stress-induced cilia ablation or damage. If so, this would trigger NRF2-dependent cytoprotective responses, which might include cilia regeneration, consistent with our data that NRF2 promotes ciliogenic gene expression (Fig. 5a, Supplementary Figs S6a and S9a). Consistent with this, NRF2 plays a protective role in cigarette smoke-induced harm to airway cilia^23^. Also, flow sensation in renal mechanosensory cilia represses mTOR to promote autophagy and reduce cell size^13,15^. Thus, it would be interesting to test whether mechanical stress in renal cilia also modulates NRF2 activity. If so, this could have important implications, as disruption of this ciliary pathway causes polycystic kidney disease (PKD), the most common ciliopathy^19^. The fact that NRF2 dysregulation is involved in kidney cystogenesis in a mouse cancer model supports a potential connection between NRF2 and PKD^49^.

Mechanistically, many questions remain concerning how the PAN axis works. Our data indicate that GLI-dependent Hh signaling is not involved (Supplementary Fig. S2), but how cilia control autophagy and NRF2 remains poorly understood. For instance, our evidence shows cilia downregulate NRF2 activity but not its protein levels (Fig. 1d,h). Since NRF2 is often regulated through protein stability^20^, this raises the question of how cilia affect NRF2 in MEFs, and whether this mechanism also applies to hESCs, where NRF2 protein levels were not assessed^12^.

We have also found that NRF2-null cells grow fewer and shorter cilia than normal in some cell types (Fig. 2, Supplementary Figs S3 and S4), and display poor Hh signaling, a cilia-dependent pathway (Fig. 3). We investigated several possible causes for these effects. Hh responsiveness in *Nfe2l2*^−/−^ MEFs was not improved by NAC, which acts in oxidatively stressed cells by replenishing their levels of glutathione, a major cellular antioxidant and ROS scavenger (Fig. 4)^40^. This largely rules out oxidative stress as a significant cause of Hh signaling defects in our model. And since Hh defects arise, at least partly, from ciliogenic defects, the lack of Hh signaling rescue by NAC also suggests, indirectly, that oxidative stress does not explain reduced ciliation in *Nfe2l2*^−/−^ MEFs. Tubastatin A, an HDAC6 inhibitor, also failed to even partially rescue Hh signaling (Fig. 4). Thus, ciliophagy, the HDAC6-dependent autophagic resorption of cilia, does not account for the Hh defects either^23^. Likewise, lack of rescue by Torin-1 shows that mTOR is not involved in poor Hh responsiveness in *Nfe2l2*^−/−^ MEFs, indicating that NRF2 affects cilia through mechanisms distinct from how cilia affect NRF2 (Fig. 4). We also failed to rescue Hh signaling with H89, a PKA inhibitor (Fig. 4). Since PKA acts as a Hh pathway repressor downstream of SMO but upstream of GLI2 and GLI3, these data pointed to Hh signaling disruption downstream of SMO and PKA^7^.

We then discovered that multiple ciliogenic and Hh pathway mRNAs are expressed at lower levels in *Nfe2l2*^−/−^ MEFs (Fig. 5a,b). These moderate but highly significant reductions in gene expression, when combined, likely account for the ciliogenic and Hh defects of *Nfe2l2*^−/−^ cells.

The evidence for this is particularly strong for the Hh pathway. GLI2 and GLI3 transcription factors are the main controllers of Hh target gene expression (as GLI1 is a Hh target gene whose expression is almost negligible before pathway stimulation)^7^. Hh signaling alters the transcriptional activities of GLI2 and GLI3, turning the former into an activator (GLI2A) and preventing the latter from turning into a repressor (GLI3R), its fate under basal conditions^7^. For these changes to occur, GLI2 and GLI3 need to accumulate at the ciliary tip, but this is strongly reduced in *Nfe2l2*^−/−^ MEFs (Fig. 6a–d)^7,44–46^. In addition, GLI2 and GLI3 protein levels are strongly reduced in these mutants (Fig. 6e,f), as are their mRNAs (Fig. 5b). These changes in GLI2 and GLI3 can readily explain the observed Hh defects. And since GLI2 and GLI3 act downstream from SMO, GPR161, IFT27 and SUFU in the Hh pathway^7^, the lower mRNA expression observed for the latter genes is likely to be of little consequence in terms of Hh pathway output (Fig. 5b). In fact, despite the lower mRNA levels in *Nfe2l2*^−/−^ MEFs, SMO protein levels and its SAG-induced cilia translocation appear unaffected in these cells (Supplementary Fig. S10c–e).

Regarding ciliogenesis, it is less clear which genes and their products are responsible for the observed defects. In principle, each of the genes studied in Fig. 5a might account for these defects, as they are all important in the process. Moreover, given that our analysis was far from exhaustive, many other ciliogenic genes might be affected in NRF2-null cells. Since IFT88 is absolutely required for ciliogenesis (Fig. 1a), we tested the role of IFT88 protein in the ciliogenic defects of *Nfe2l2*^−/−^ MEFs. Intriguingly, neither total nor ciliary IFT88 protein levels were visibly affected in mutant relative to control MEFs (Supplementary Fig. S10a,b). This might indicate that compensatory mechanisms operate in *Nfe2l2*^−/−^ MEFs to keep IFT88 and SMO protein levels constant, despite lowered mRNA levels.

Some RFX transcription factors promote ciliogenesis by stimulating expression of ciliogenic genes such as the ones in Fig. 5a, so a reduction in one or more pro-ciliogenic RFX factors could neatly explain why NRF2-null MEFs grow fewer and shorter cilia. RFX1-4 are well established pro-ciliogenic factors^4^, but none of them was reduced in *Nfe2l2*^−/−^ MEFs. Instead, we found reductions in *Rfx5* and *Rfx7* (Supplementary Fig. S7a). RFX5 and RFX7 constitute a distinct RFX subfamily and both play important roles in immunity^4,5,50^, yet only RFX7 has recently been linked to ciliogenesis in the neural tube^51^. Whether RFX7 contributes to the ciliogenic phenotype of NRF2-null cells remains unaddressed.

Unlike *Rfx5* and *Rfx7*, we found elevated levels of *Rfx4* and *Rfx8* in NRF2-null MEFs. This, however, cannot explain the ciliogenic defects, which were seen in both primary and immortalized *Nfe2l2*^−/−^ MEFs (Fig. 2a–c and Supplementary Fig. S3), whereas *Rfx4* upregulation only occurred in the former (Supplementary Fig. S7b). Additionally, higher RFX4 levels would be expected to upregulate ciliogenic genes, not reduce them. Virtually nothing is known about RFX8, except that it belongs to the same RFX subfamily as RFX4^4,5^.

Since NRF2 stimulates its target genes by binding to ARE sequences in their promoters, we performed a bioinformatic search for putative ARE sequences in the human promoters of the ciliogenic and Hh pathway genes we found reduced in *Nfe2l2*^−/−^ MEFs (Fig. 5c). Remarkably, the top two hits from this analysis were *GLI2* and *GLI3*, whose promoters each contain a perfect match with the consensus ARE sequence (relative score = 0.997). Together with the above data, this suggests that *GLI2* and *GLI3* may be direct NRF2 targets, although this awaits experimental demonstration. Putative ARE sequences with relative scores in the 0.81–0.98 range were also found in *IFT172*, *IFT140*, *IFT88*, *IFT74*, *GPR161*, *PTCH1* and *IFT27*. Consistent with these data, *Ift74*, *Ift27* and *Gpr161* promoter sequences were pulled down with NRF2 in a ChIP-seq study in sulforaphane-treated cells^52^. The presence of putative AREs in *PTCH1* (but not *GLI1*) promoter raises the possibility that NRF2 directly upregulates Hh targets. If so, we would expect lower basal *PTCH1* levels in unstimulated NRF2-null MEFs, which we did not detect. However, since basal *PTCH1* levels are very low, detecting such a reduction, if it exists, could be very challenging. Using cells with higher basal *PTCH1* levels could clarify this issue.

Since genetic deletion of NRF2 reduces ciliogenic and Hh pathway gene expression, we also tested whether NRF2 stabilization by DMF had the opposite effect. Interestingly, ciliogenic genes *Dync2h1* and *Ift88* were both significantly upregulated in DMF-treated *Nfe2l2*^+/+^ but not *Nfe2l2*^−/−^ MEFs (Supplementary Fig. S9a). This confirms these genes are positively regulated by NRF2. For *Gli2*, *Gli3* and *Smo*, although the data pointed in the same direction, DMF did not cause any statistically significant changes (Supplementary Fig. S9b). Likewise, DMF did not increase ciliogenesis or Hh responsiveness above basal levels, indicating that NRF2 levels in these cells are not limiting for these processes (Supplementary Fig. S8).

Beyond the mechanistic details, our work raises an important question: what is the biological significance of NRF2 affecting ciliogenesis and Hh signaling? Severe ciliogenesis and Hh signaling disruptions in mouse embryos lead to embryonic lethality and malformations^7,53^. In contrast, *Nfe2l2*^−/−^ mice are viable, have no malformations, and do fairly well unless exposed to stress^31,54^. Hence, cilia and Hh signaling cannot be severely perturbed in *Nfe2l2*^−/−^ mouse embryos. Together with our data, this suggests that NRF2 affects ciliogenesis and Hh signaling in a manner dependent on cell type, tissue and developmental stage. Comprehensive spatiotemporal studies would shed light on this issue.

Our data show NRF2 affects ciliogenesis in embryonic fibroblasts and hippocampal astrocytes, so one might expect NRF2-null mice to have cilia or Hh-dependent phenotypes related to these cell types. In this regard, we recently reported defects in hippocampal neurogenesis in NRF2-null mice, a process where cilia and Hh signaling also play important roles^32,55–57^. Thus, neurogenesis defects in NRF2-null hippocampus may be related to defective ciliation. If so, this might involve astrocyte cilia, but maybe also neuronal cilia, which are not labeled by ARL13B and thus were not visualized in our study^37,38^. On the other hand, we saw no ciliary defects in multiciliated ependymal cells of NRF2-null brain ventricles (Supplementary Fig. S4). This suggests that cilia defects in NRF2-null mice are cell type-specific, thereby explaining why these animals do not suffer from hydrocephalus, a pathology resulting from ependymal cilia malfunction^4,31^.

Why NRF2 affects ciliogenesis in some cell types more than others remains a mystery. Cell type-specific compensatory mechanisms may exist, which could act at many levels, such as gene expression, protein stability or enzyme activity, to name a few. Redundancy is another possibility: NFE2L1/NRF1, an NRF2-related transcription factor, acts redundantly with NRF2 during mouse development^58^. Thus, our data might be explained by NRF1 compensating for NRF2 loss in specific cell types. If so, one would expect *Nfe2l1-Nfe2l2* double knockout mice to display cilia and Hh-related phenotypes. These embryos are delayed, show signs of massive oxidative stress and apoptosis, and die at midgestation, too early to assess for all but the most drastic defects in cilia and Hh signaling^1,7,58^. On the other hand, NRF1 is expressed ubiquitously, and *Nfe2l1-Nfe2l2* double mutant MEFs show markedly higher apoptosis and stress sensitivity than *Nfe2l2*^−/−^ MEFs, demonstrating that NRF1 is present and functional in the latter^58,59^. Therefore, lack of NRF1 function is unlikely to explain cilia and Hh defects in *Nfe2l2*^−/−^ MEFs. Still, factors other than NRF1 might act redundantly with NRF2.

Another possibility is that Hh signaling participates in NRF2-dependent cytoprotective responses. Since NRF2 promotes Hh signaling, stress-induced NRF2 stabilization might enhance Hh signaling, which might protect cells from damage in some way. Although Hh signaling stimulation by oxidative stress and cytoprotective effects of Hh signaling are both reported in the literature^60–65^, our data showed no enhancement of Hh responsiveness by DMF, as mentioned above (Supplementary Fig. S8c). This, however, does not rule out that Hh responses play cytoprotective roles *in vivo*, particularly in cell types where basal NRF2 protein levels are low relative to what cells need for optimal Hh responsiveness.

Here, we have established a clear connection between NRF2, expression of ciliogenic and Hh pathway genes, and the processes controlled by these genes. The physiopathological contexts where changes in NRF2 activity affect cilia and Hh signaling remain to be elucidated.

## Methods

### Reagents, plasmids and antibodies

*Reagents*: Torin-1 (ApexBio, 250 nM), Rapamycin (Fisher Bioreagents, 200 nM), dimethyl fumarate (Sigma, 20 µM), Chloroquine (Acros Organics, 10 µM), SAG (Cayman, 200 nM), R,S-Sulforaphane (LKT Laboratories, 15 µM), N-acetyl-L-cysteine (Alfa Aesar, 3 mM), H89 dihydrochloride (BioVision, 10 µM) and Tubastatin A (Cayman, 10 µM). *Plasmids*: pcDNA3.1-mGli2ΔN and pcDNA3.1-hGLI3R were kind gifts of Drs. Victor L. Ruiz-Perez and Elisa Martí, respectively^66^. *Antibodies*: Acetylated α-tubulin (Sigma, T7451, 1:10,000), GLI1 (Cell Signaling, L42B10, 1:1000), GFAP (Sigma, G3893, 1:200), HMOX1 (Enzo Life Sciences, 1:2000), SMO (Proteintech, 66851-1-Ig, WB: 1:500), GAPDH (Proteintech, 60004-1-Ig, 1:1000), β-actin (Proteintech, 66009-1-Ig, 1:5000) and α-tubulin (Proteintech, 66031-1-Ig, 1:1000) were from mouse. GLI2 (R&D systems, AF3635, WB: 0.1 µg/ml, IF: 4 µg/ml), GLI3 (R&D systems, AF3690, WB: 1 µg/ml, IF: 4 µg/ml), Lamin B (Santa Cruz, sc-6217, 1:5000) and γ-tubulin (Santa Cruz, sc-7396, 1:200) were from goat. ARL13B (Proteintech, 17711-1-AP, 1:200), LC3B (Cell Signaling Technology, #2775, 1:500), SMO (Abcam, ab38686, IF: 1:1000), IFT88 (Proteintech, 13967-1-AP, WB: 1:1000, IF: 1:50), CEP164 (Proteintech, 22227-1-AP, 1:500) and NRF2 (described in^21^, 1:5000) were from rabbit. Due to their initial low specificity (see Fig. 3b and Supplementary Fig. S12), NRF2 antibodies were repeatedly incubated with membranes containing NRF2-null cell lysates, which led to greater specificity towards NRF2, as seen in Fig. 1d,h and Supplementary Fig. S12. Secondary antibodies were from donkey (Thermofisher, AlexaFluor 488, 555 or 647-conjugated) or from goat (Thermofisher, HRP-conjugated).

### Animals

Colonies of *Nfe2l2*^+/+^ and *Nfe2l2*^−/−^ C57BL/6 mice were established from founders kindly provided by Prof. Masayuki Yamamoto^67^. Animal procedures were performed according to protocols approved by the Ethical Committee for Research of the Spanish National Research Council (CSIC), in accordance with institutional, Spanish and European guidelines (*Boletín Oficial del Estado*, 18 March 1988; and European Council Directives 86/609/EEC and 2003/65/EC).

### Cell culture

*Kif3a*^+/+^, *Kif3a*^−/−^, *Ift88*^+/+^ and *Ift88*^−/−^ MEFs have been reported elsewhere^68^. *Nfe2l2*^+/+^ and *Nfe2l2*^−/−^ littermate MEFs were derived from E11.5 mouse embryos and immortalized with SV40 large T antigen using previously described protocols^43^. All MEFs were grown in DMEM medium supplemented with 10% fetal bovine serum at 37 °C and 5% CO_2_ in a humidified atmosphere. All cell lines were mycoplasma-free, as ascertained by regular tests. To induce ciliogenesis and for drug treatments, MEFs were starved for 24 hours in OptiMEM medium (Thermofisher). For autophagy flux analysis, cells were starved for 4 hours in Earle’s Balanced Salts Solution (EBSS, Sigma).

### RT-qPCR

Total cellular RNA was extracted using GeneJET RNA purification kit (Thermofisher) and reverse transcribed with MMLV Reverse Transcriptase (Promega) using oligo-dT_18_. Quantitative real time PCR was performed using PowerUp SYBR Green Master Mix (Thermofisher) and the primer pairs listed in Supplementary Fig. S11. Reactions were run on an Applied Biosystems StepOne Real Time PCR System and data analyzed using ΔΔC_t_ method with β-actin as control gene.

### Western blot and quantitation

Total cell lysates were prepared in buffer containing 50 mM Tris-HCl pH 7.5, 150 mM NaCl, 1% Igepal CA-630 and protease inhibitor cocktail (Thermofisher). Lysates were centrifuged at 20,000 × g for 10 min at 4 °C to obtain postnuclear supernatants, whose protein concentrations were equalized after measuring them with Pierce BCA Protein Assay kit. Samples were then processed for SDS-PAGE, run in 4–20% Novex Tris-Glycine gels (Thermofisher) and transferred to nitrocellulose membranes for immunostaining. Results were visualized using ECL chemiluminescence reagents and captured on either X-ray film or using an MF-ChemiBIS 3.2 system (DNR Bio-Imaging Systems). Images were then cropped and their brightness and contrast adjusted using Adobe Photoshop. Uncropped and unadjusted images are shown in Supplementary Fig. S12. For band quantitation, Fiji (Image J) software was used. Briefly, 8-bit grayscale images of blots were inverted and average pixel intensity measured for all bands of interest and their corresponding backgrounds. Total pixel intensity of each band was then obtained by multiplying average pixel intensity by pixel number. To this value, total pixel intensity of an equally-sized background area was subtracted in order to obtain the specific band signal, which was expressed as a ratio to the control band. For autophagy flux calculation, basal LC3B-II/LC3B-I ratio was subtracted from the same ratio in presence of chloroquine, and the resulting difference was normalized relative to control sample.

### Immunofluorescence and quantitation

Cultured cells were grown on coverslips, fixed 5 min at RT in PBS with 4% paraformaldehyde, then 3 min at −20 °C in freezer-cold methanol. Cells were blocked and permeabilized in blocking solution (PBS + 0.1% Triton X100 + 2% donkey serum + 0.02% sodium azide) for 30–60 min at RT before proceeding with standard immunostaining procedures and imaging with a Nikon 90i fluorescence microscope. All cilia quantitations were performed with the aid of Fiji (Image J) software. For percent ciliation, one or more coverslips per condition were examined in each independent experiment. For each coverslip, seven representative cell fields were used to count cilia (identified by ARL13B and γ-tubulin costaining), whose number was divided by total cells (DAPI-labeled nuclei). For length measurements, cilia were identified as above and their length measured in the ARL13B channel. For ciliary intensity measurements, unsaturated 8-bit images were used. Total signal in the region of interest (ciliary tip region for GLI2-3 or whole cilium for IFT88 and SMO) was obtained for the relevant channel as the product of average pixel intensity and pixel number in the region. Background signal was likewise measured near each region of interest and subtracted from total signal to obtain the specific signal. For *in vivo* studies, 30 µm-thick coronal brain sections of 3-month-old mice were obtained and processed as previously described^32^, and imaged using a Leica TCS SP5 confocal microscope. Ependymal cilia were visualized in one mouse of each genotype. For quantitation of hippocampal samples, five sections were imaged for each of three mice. Fiji was then used to count cilia, identified as rod-shaped ARL13B^+^ structures, and GFAP^+^ astrocytes. Since ARL13B^+^ cilia were only seen in close association with astrocytes (consistent with reports indicating that ARL13B is an astrocytic ciliary marker in adult brain^37,38^), we plotted data as percentage of GFAP^+^ astrocytes carrying ARL13B^+^ cilia.

### Bioinformatics

Putative ARE sequence identification was performed as previously described^22^. Briefly, we first identified putative AREs in the promoter regions of our genes of interest using the Encyclopedia of DNA Elements at UCSC (ENCODE) of the human genome (Feb. 2009), based on the ChIP data available for ARE-binding transcription factors BACH1 and MAFK. Subsequently, using a Python-based script, these putative AREs were compared with the position-specific scoring matrix (PSSM) derived from the frequency matrix of the consensus ARE sequence recognized by NRF2, obtained from JASPAR database. We considered only putative AREs with a relative score higher than 80%^22^.

### Statistical analysis

GraphPad Prism 7 software was used to graph and statistically analyze data. The specific details of each experiment are provided in the corresponding figure legends.

## Supplementary information

Supplementary Figures S1-S12

## Data Availability

The datasets generated during the current study are available from the corresponding author on reasonable request.
