# Supplementary material for: NRF2-dependent gene expression promotes ciliogenesis and Hedgehog signaling
Source: Sci Rep. 2019 Sep 25;9:13896. doi: 10.1038/s41598-019-50356-0 (PMC6761261; doi:10.1038/s41598-019-50356-0)
Supplement: Supplementary file 1 — Supplementary Figures S1-S12 [file 41598_2019_50356_MOESM1_ESM.pdf]

SUPPLEMENTARY INFORMATION

NRF2-dependent gene expression promotes ciliogenesis and Hedgehog signaling

Ana Martin-Hurtado, Raquel Martin-Morales, Natalia Robledinos-Antón, Ruth Blanco, Ines Palacios-Blanco, Isabel Lastres-Becker, Antonio Cuadrado & Francesc R. Garcia-Gonzalo

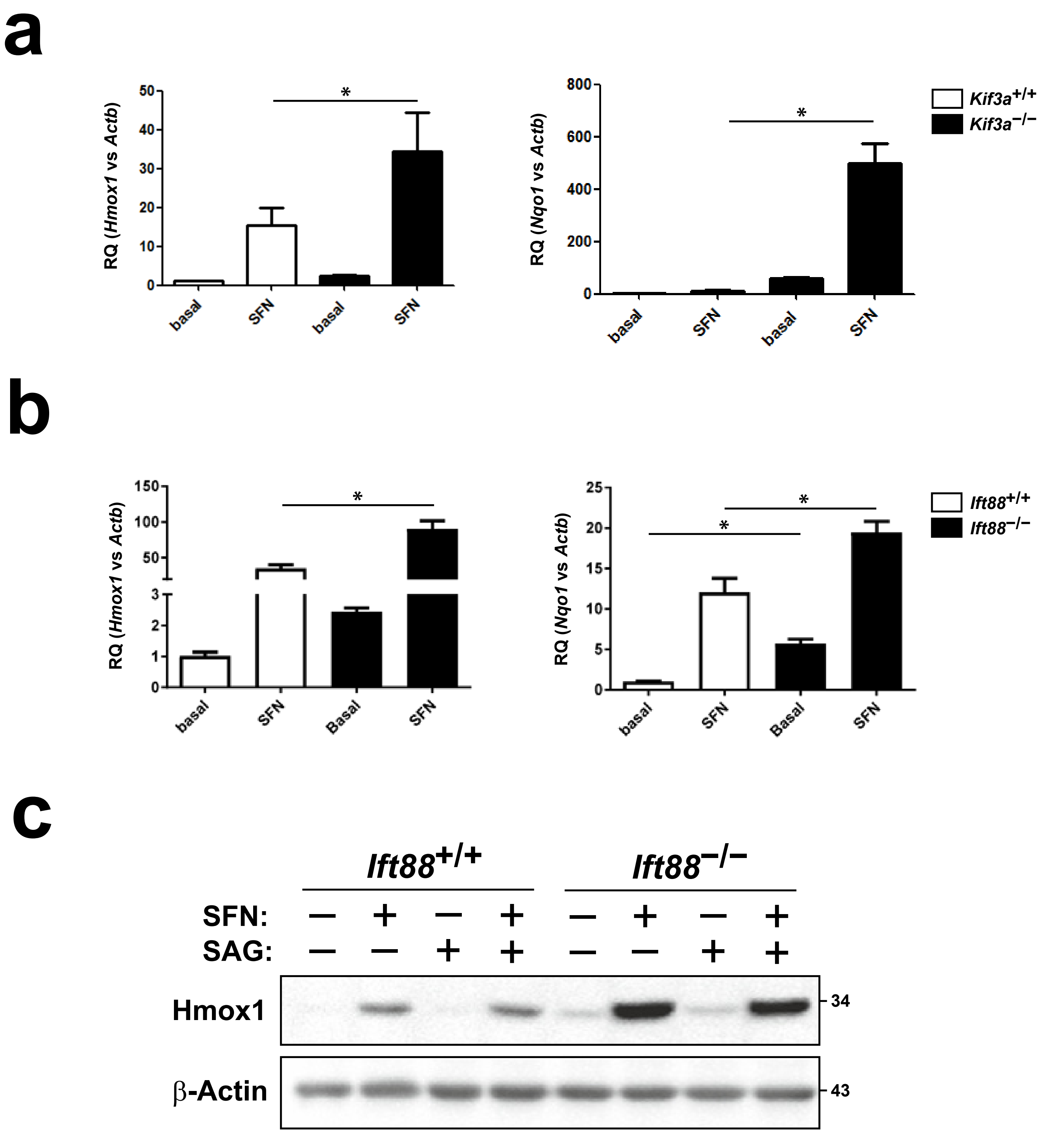

Supplementary Figure S1. Cilia downregulate NRF2 transcriptional activity. **(a)** *Kif3a*$^{+/+}$ and *Kif3a*$^{-/-}$ littermate MEFs were starved 24h with or without 15μM Sulforaphane (SFN) and expression levels of NRF2 target genes *Hmox1* and *Nqo1* were analyzed by RT-qPCR. **(b)** Same experiment as in a was performed with *Ift88*$^{+/+}$ and *Ift88*$^{-/-}$ littermate MEFs. Data in a and b are means ± SEM of three technical replicates. Asterisks indicate significance in Student's t-test with $p<0.05$. **(c)** *Ift88*$^{+/+}$ and *Ift88*$^{-/-}$ MEFs were treated 24h with 15μM SFN and/or 200nM SAG, as indicated, and protein levels of Hmox1 were assessed by Western blot using β-actin as loading control. Molecular weight markers are shown on the right.

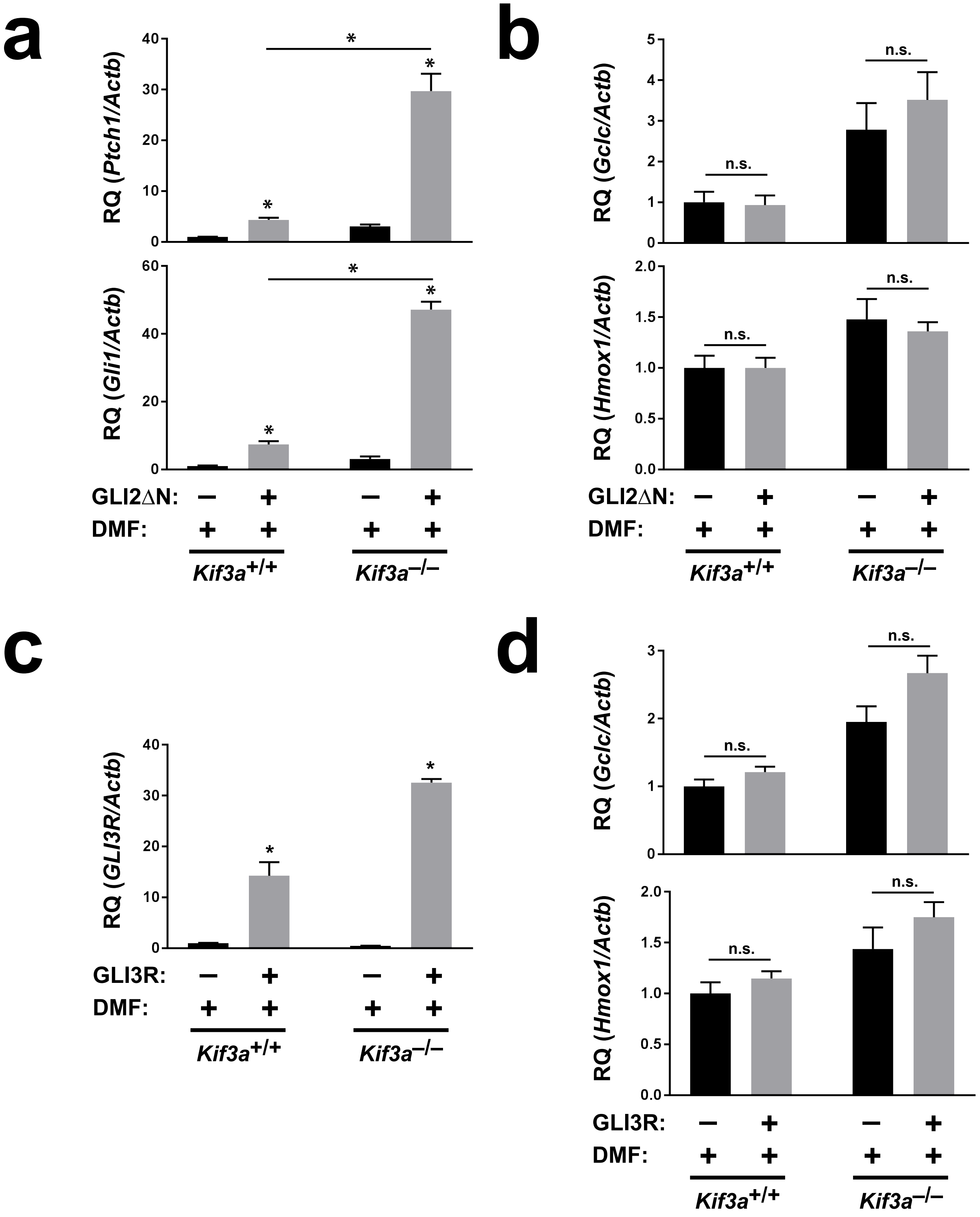

Supplementary Figure S2. Hh signaling is not involved in ciliary downregulation of NRF2 activity. **(a)** *Kif3a*$^{+/+}$ and *Kif3a*$^{-/-}$ MEFs were transfected with empty vector or plasmid expressing the constitutively active GLI2ΔN mutant. Cells were starved 24h in presence of 20μM DMF and gene expression of the indicated Hh target genes was measured by RT-qPCR. **(b)** Expression of the indicated NRF2 target genes was measured in the experiment described in a. **(c)** Experiment as in a-b, except that cells were transfected with empty vector or plasmid expressing GLI3R, whose expression was assessed by RT-qPCR. **(d)** Expression of the indicated NRF2 target genes was measured in the experiment described in c. In all cases, β-actin (*Actb*)-normalized relative mRNA levels are displayed as mean ± SD of four technical replicates. Asterisks indicate significance in a Student's t-test with $p<0.05$ (n.s.: not significant).

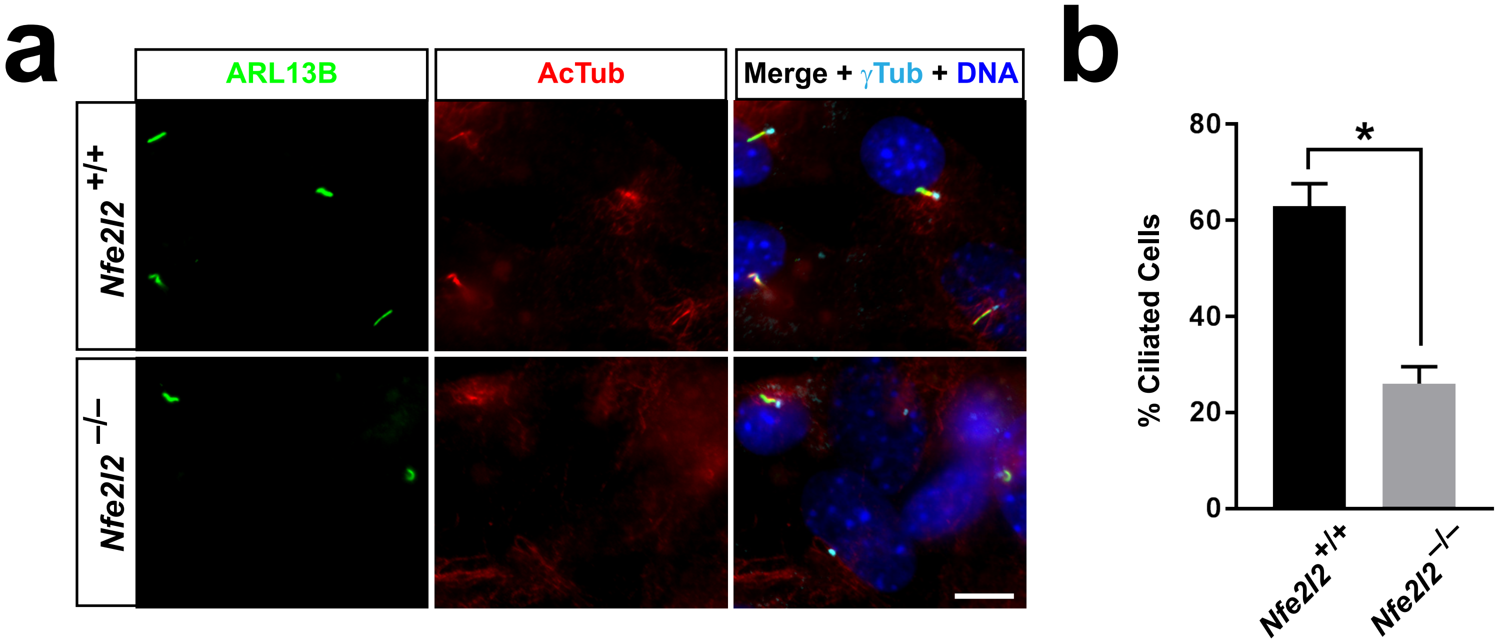

Supplementary Figure S3. Ciliogenesis is reduced in NRF2-null primary MEFs. **(a)** Representative immunofluorescence images of ARL13B, acetylated α-tubulin (AcTub) and γ-tubulin (γTub) in *Nfe2l2*$^{+/+}$ and *Nfe2l2*$^{-/-}$ primary MEFs starved 24h to induce ciliogenesis. Nuclei were counterstained with DAPI (DNA). Scale bar, 10μm. **(b)** Quantitation of percentage of primary MEFs from (a) harboring ARL13B^{+} cilia. Data are mean ± SEM of n=3 independent experiments. Asterisk indicates significance with p<0.05 according to unpaired two-tailed t-test.

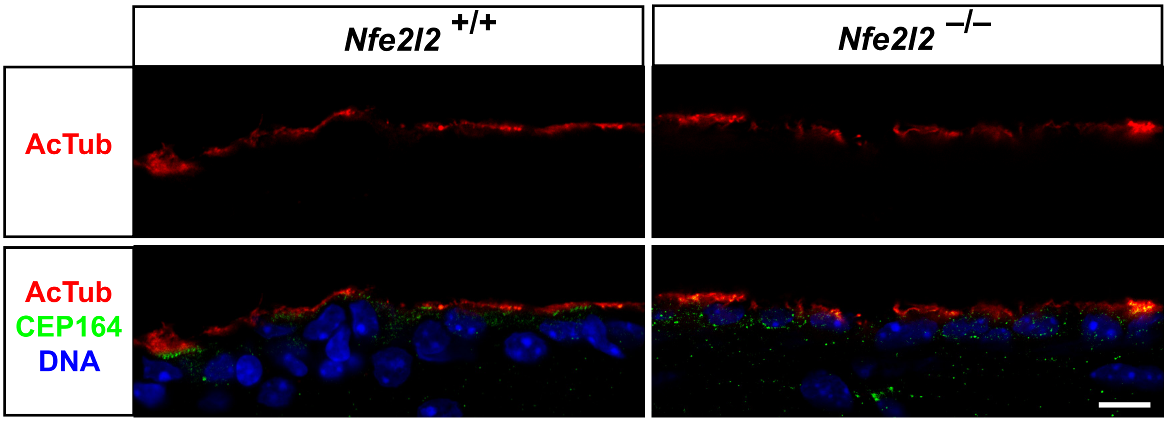

Supplementary Figure S4. Ciliogenesis appears normal in NRF2-null brain ventricles. Representative immunofluorescence images of acetylated α-tubulin (AcTub) and CEP164 in the brain ventricles of *Nfe2l2*$^{+/+}$ and *Nfe2l2*$^{-/-}$ mice showing multiciliated ependymal cells. Nuclei were counterstained with DAPI (DNA). Images are single confocal sections. Scale bar, 10μm.

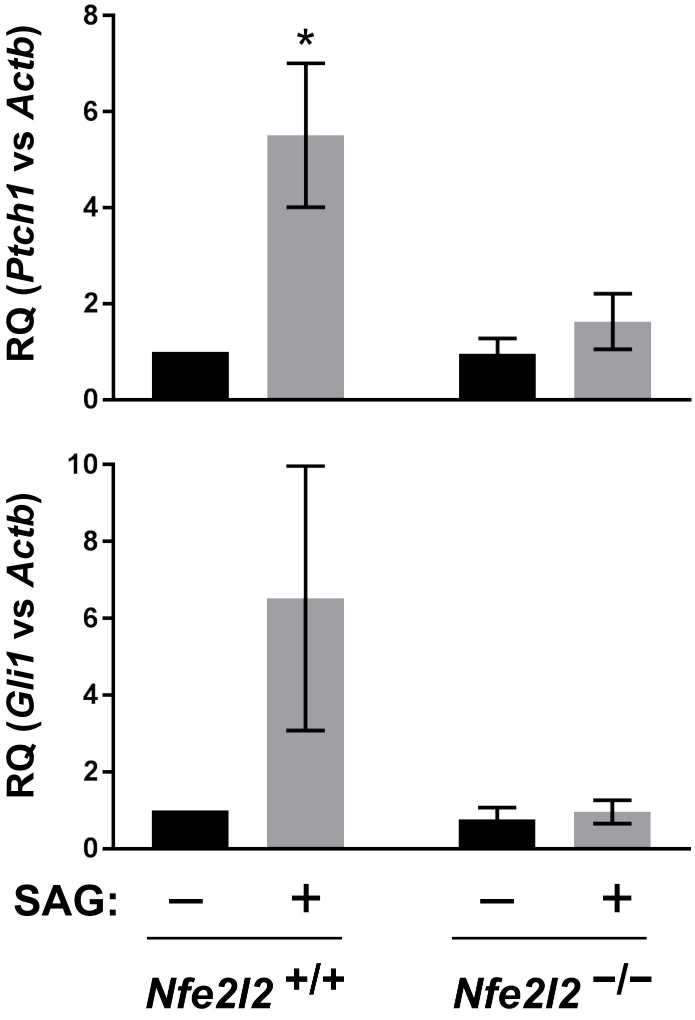

Supplementary Figure S5. Hh responsiveness is reduced in NRF2-null primary MEFs. *Nfe2l2*$^{+/+}$ and *Nfe2l2*$^{-/-}$ primary MEFs were starved 24h in presence of DMSO (vehicle) or SAG (200nM) and gene expression of *Ptch1* (top) and *Gli1* (bottom) were analyzed by RT-qPCR. Data are displayed as β-actin-normalized relative mRNA levels (mean ± SEM, n=3 independent experiments). Asterisk indicates significance ($p<0.05$) in unpaired two-tailed t-test.

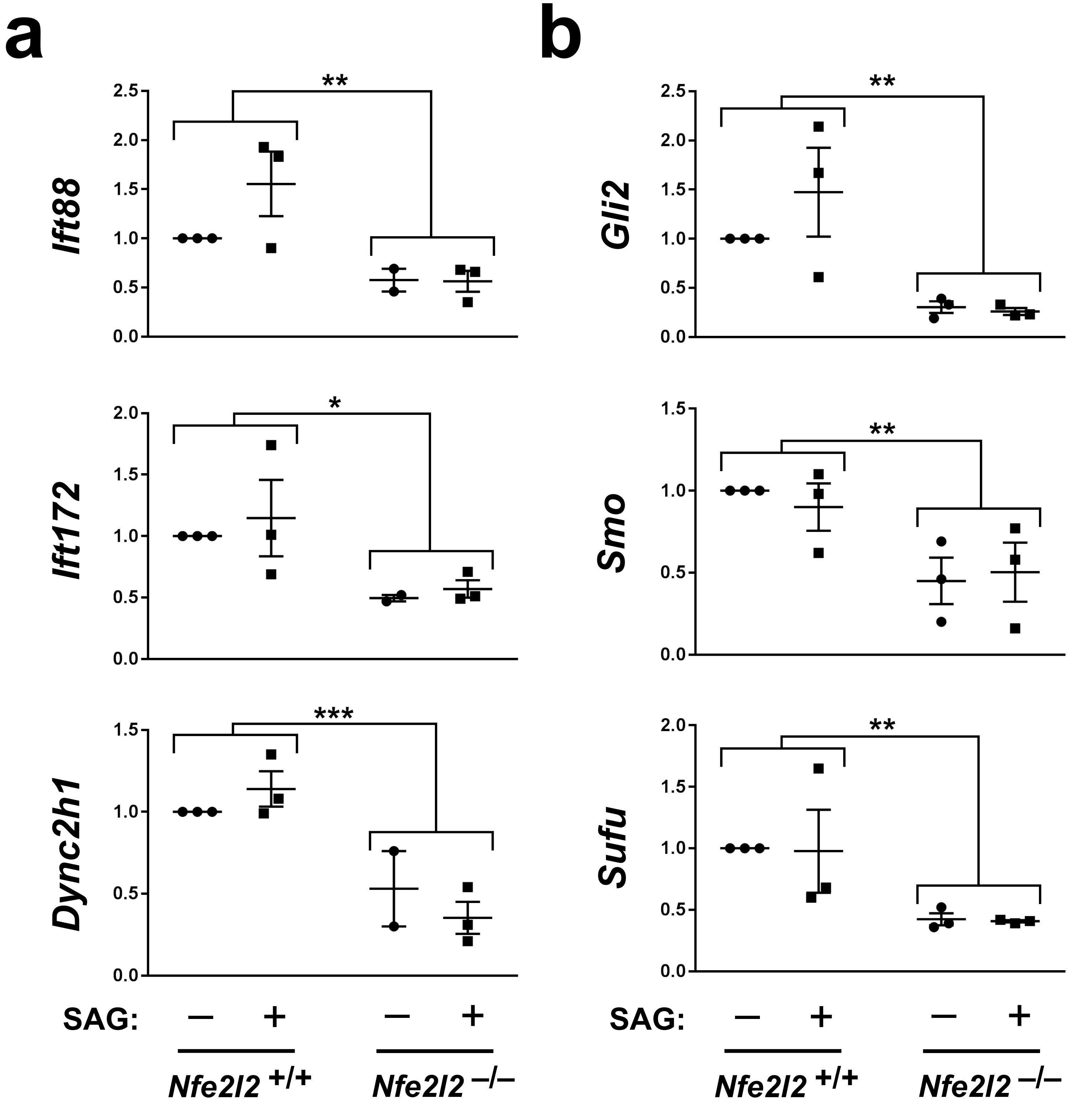

Supplementary Figure S6. Ciliogenic and Hh pathway gene expression is reduced in NRF2-null primary MEFs. (a-b) *Nfe2l2* $^{+/+}$ and *Nfe2l2* $^{-/-}$ primary MEFs were starved 24h in presence of DMSO or 200nM SAG and gene expression of the indicated ciliogenic (a) or Hh pathway (b) genes was analyzed by RT-qPCR. Data are displayed as β-actin-normalized relative mRNA levels (mean ± SEM , n=3 independent experiments). Asterisks indicate significant differences between genotypes in two-way ANOVA with $p<0.05$ (*), $p<0.01$ (**) and $p<0.001$ (***).

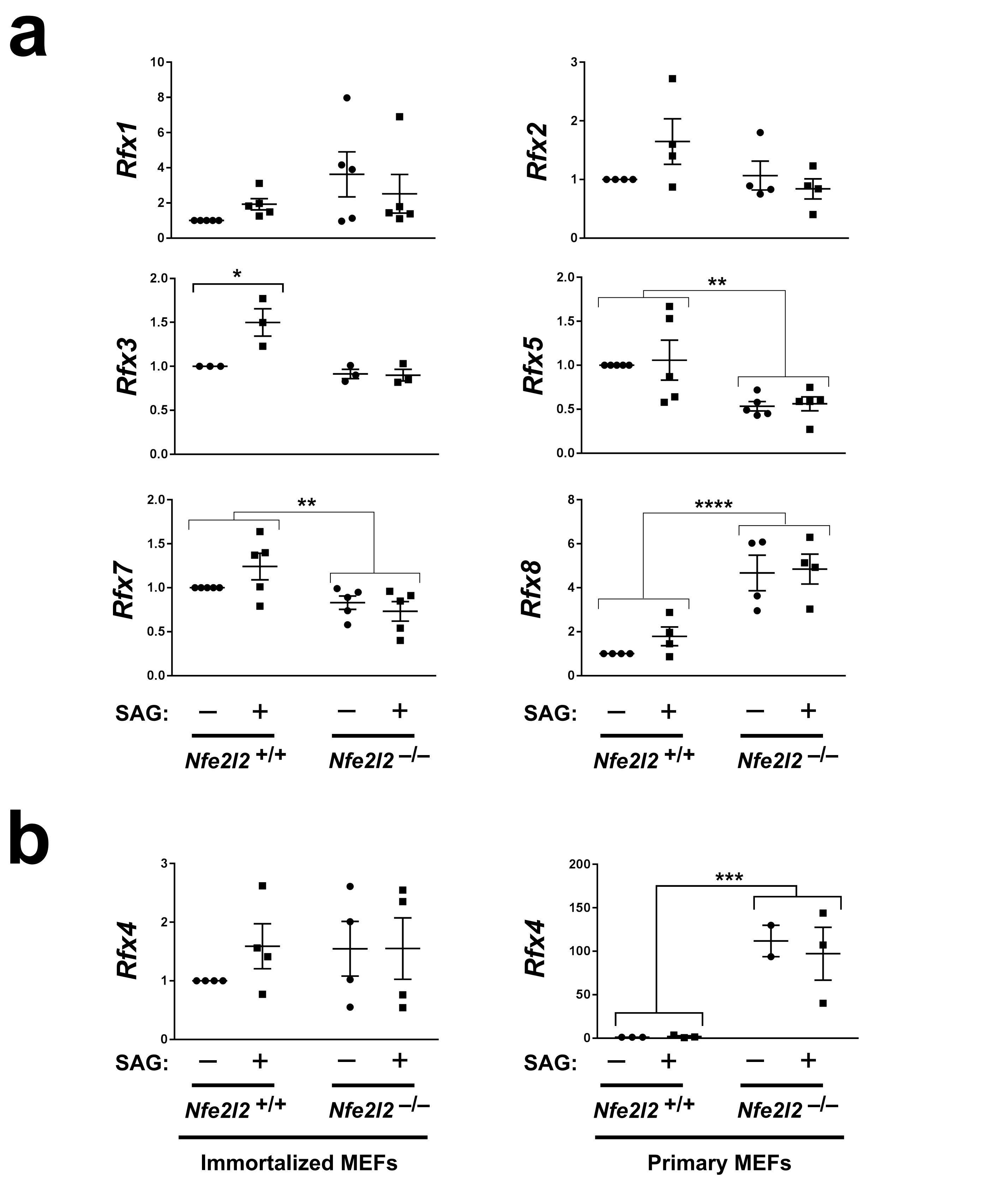

Supplementary Figure S7. RFX gene expression is altered in NRF2-null cells. **(a)** *Nfe2l2* $^{+/+}$ and *Nfe2l2* $^{-/-}$ immortalized MEFs were starved 24h in presence of DMSO or SAG (200nM) and expression of the indicated *Rfx* genes was analyzed by RT-qPCR. **(b)** Same experiment as in a was performed for *Rfx4* in both immortalized or primary *Nfe2l2* $^{+/+}$ and *Nfe2l2* $^{-/-}$ MEFs, as indicated. All data are shown as β-actin-normalized relative mRNA levels (mean ± SEM, n=3-5 independent experiments). Statistical analysis: two-way ANOVA followed by Tukey's multiple comparisons tests. Significance: p<0.05 (*), p<0.01 (**), p<0.001(***) or p<0.0001 (****).

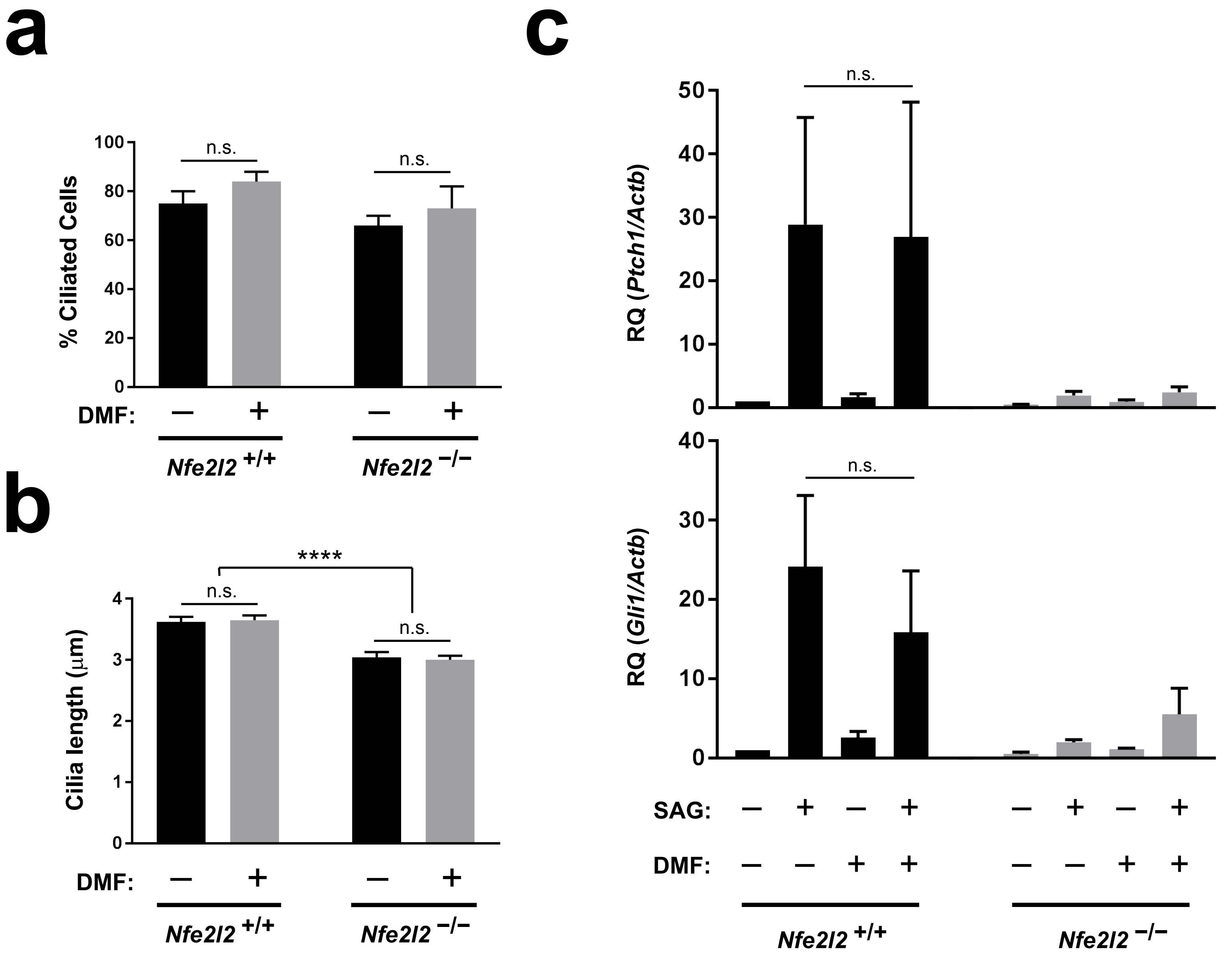

Supplementary Figure S8. DMF does not enhance ciliogenesis or Hh responsiveness. **(a)** *Nfe2l2*$^{+/+}$ and *Nfe2l2*$^{-/-}$ MEFs were starved 24h in presence or absence of 20μM DMF, their cilia stained by immunofluorescence as in Fig.2a, and percentage of ciliated cells quantitated as in Fig.2b. Data are mean ± SEM of n=7 fields of cells per condition in a single experiment. **(b)** Quantitation of cilia length from experiment in a was performed as in Fig.2c. Data are mean ± SEM of n=77-98 cilia per condition. **(c)** *Nfe2l2*$^{+/+}$ and *Nfe2l2*$^{-/-}$ MEFs were starved 24h in presence of DMSO, 200nM SAG and/or 20μM DMF, as indicated, and expression of Hh target genes *Ptch1* (top) and *Gli1* (bottom) was analyzed by RT-qPCR. Data shown as β-actin (*Actb*)-normalized relative mRNA levels (mean ± SEM of n=3 independent experiments). DMF did not cause any significant differences. **(a-c)** Statistical analysis: two-way ANOVA followed by Tukey's multiple comparisons tests. Asterisks: p<0.0001(****); n.s.: not significant.

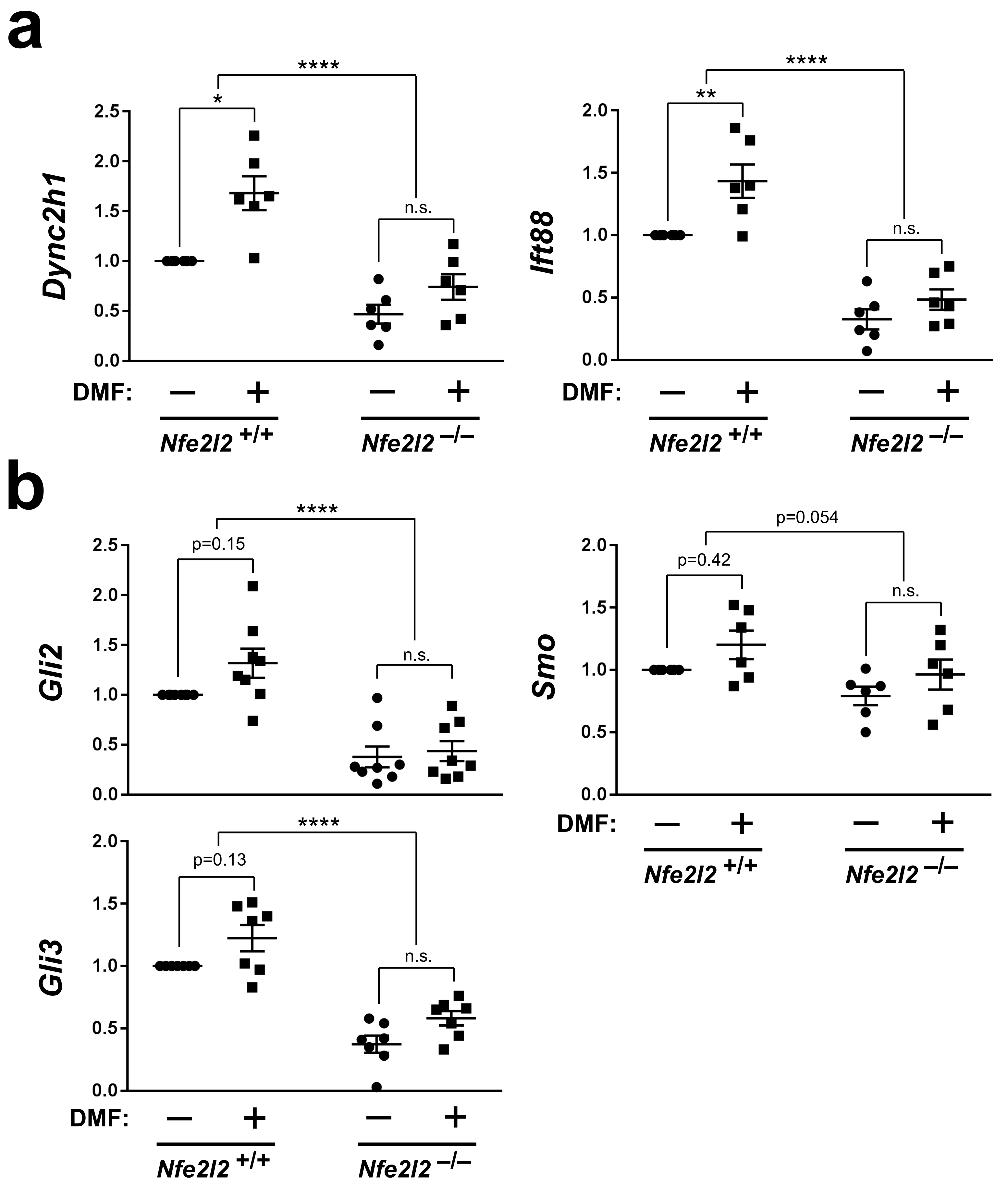

Supplementary Figure S9. Effect of DMF on ciliogenic and Hh pathway gene expression. **(a)** *Nfe2l2*$^{+/+}$ and *Nfe2l2*$^{-/-}$ MEFs were starved 24h in presence or absence of 20μM DMF and gene expression of ciliogenic genes *Dync2h1* and *Ift88* was analyzed by RT-qPCR. **(b)** Same was done for Hh pathway genes *Gli2*, *Gli3* and *Smo*. All data are β-actin-normalized relative mRNA levels and are displayed as mean ± SEM of n=6-8 independent experiments. Statistical analysis: two-way ANOVA followed by Tukey's multiple comparisons tests. Asterisks: $p<0.05$ (*), $p<0.01$ (**), $p<0.0001$ (****) (n.s.: non-significant).

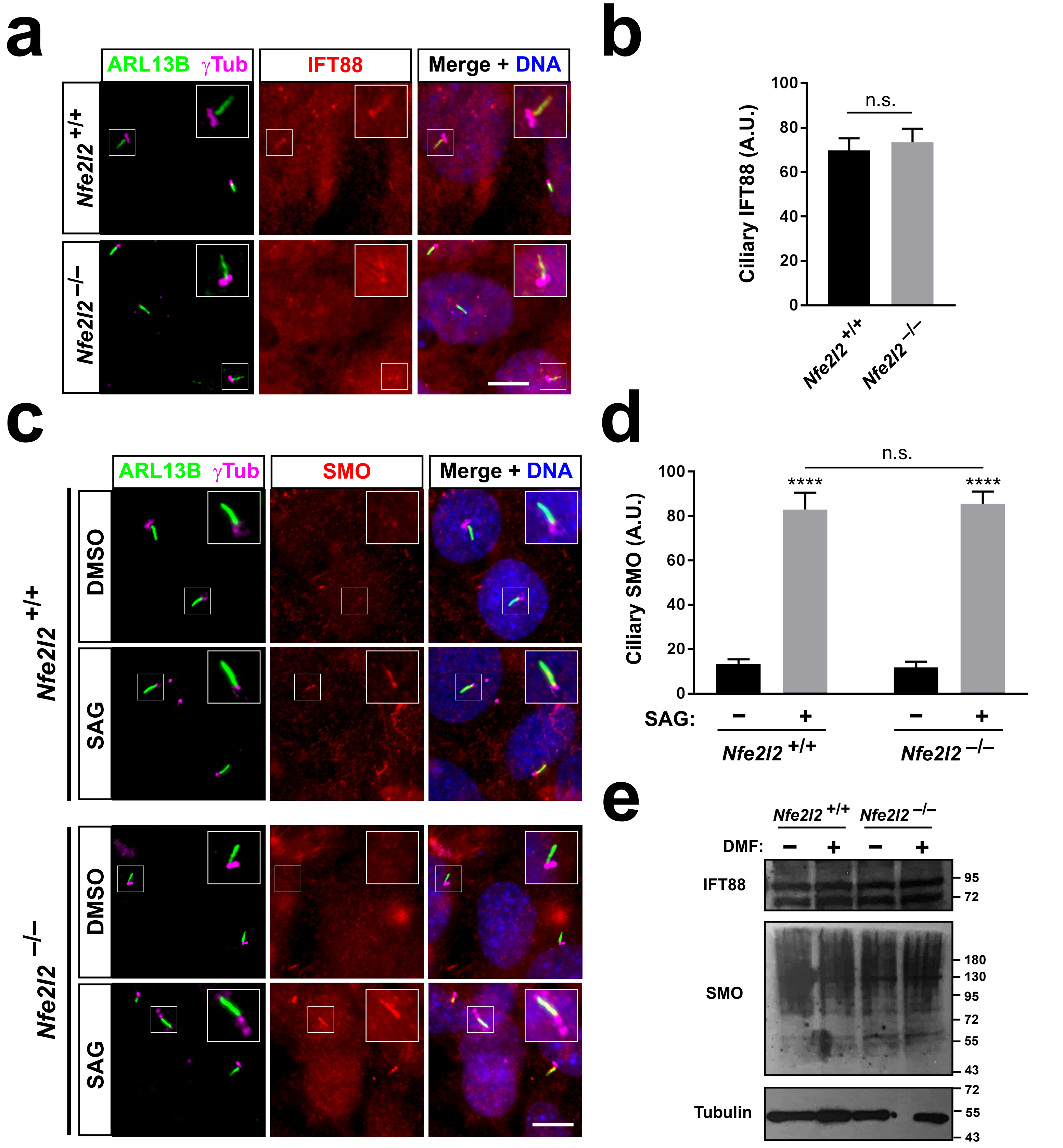

Supplementary Figure S10. IFT88 and SMO cilia localization and protein levels are unaffected in NRF2-null cells. **(a)** Representative immunofluorescence images for ARL13B, γ-tubulin and IFT88 in *Nfe2l2*$^{+/+}$ and *Nfe2l2*$^{-/-}$ MEFs serum-starved for 24h. Nuclei were counterstained with DAPI. Top right of each image shows magnification of boxed area. Scale bar, 10μm. **(b)** Quantification of IFT88 ciliary intensity from a. Data are mean ± SEM of n>33 cilia per condition (A.U.: arbitrary units). Data are not significant (n.s.) according to unpaired t-test. **(c)** Same analysis as in a was performed for SMO in presence of either DMSO (vehicle) or SAG (200nM). Scale bar, 10μm. **(d)** Quantification of SMO ciliary intensity from c was carried out as in b. Data are mean ± SEM of n>55 cilia per condition. Statistical analysis: two-way ANOVA followed by Tukey's multiple comparisons tests. Asterisks indicate p<0.0001 (****) relative to vehicle. **(e)** *Nfe2l2* $^{+/+}$ and *Nfe2l2* $^{-/-}$ MEFs were starved for 24h in presence or absence of DMF and analyzed by Western blot with IFT88 and SMO antibodies. Alpha-tubulin was used as loading control. Molecular weight markers in kDa are on the right.

Gene	Primer Name	Primer Sequence
Hmox1	mHmox1_F	CACAGATGGCGTCACTTCGTC
	mHmox1_R	GTGAGGACCCACTGGAGGAG
Gclc	mGclc_F	CCCAGACTAGGCTGTCCTGGATTCAC
	mGclc_R	GACAGCAGTTGCCCATCCCGAATCC
Actb	mActb_F	CACAGCTTCTTTGCAGCTCCTT
	mActb_R	CGTCATCCATGGCGAACTG
Ptch1	mPtch1_F	CTCTGGAGCAGATTTCCAAGG
	mPtch1_R	TGCCGCAGTTCTTTTGAATG
Gli1	mGli1_F	GGTGCTGCCTATAGCCAGTGTCCTC
	mGli1_R	GTGCCAATCCGGTGGAGTCAGACCC
Ift74	mIft74_F	AAAGTTGCTGATCGTCCTGTGACCC
	mIft74_R	ACATGTTGTAGTCTGCTAGTTGTCC
Ift88	mIft88_F	GTCCTGGTGAGACAACGAGAGC
	mIft88_R	TGGGATCTGATCTAAGGCCATTCG
Ift140	mIft140_F	ACTGACATTGCCATCCTCAGTTGG
	mIft140_R	ATCCATGAGACTGACAAAGAAAGACAGC
Ift172	mIft172_F	GAGACAAGTTCTCCACCAAACCAGC
	mIft172_R	AGGCCACTGCAGACAAGTCACAGC
Dync2h1	mDync2h1_F	CAGTACTCCCATGTCTTGGAACGG
	mDync2h1_R	CTCTGTCTAGCTGACTAATGAGGACC
Rfx1	mRfx1_F	CCTGTGGCTCAGCAGTATATCGTGG
	mRfx1_R	CTGCACACTTGCCTGGACCAGCAGC
Rfx2	mRfx2_F	TCTCTGGCCCACACTGCACGATCC
	mRfx2_R	TGCGGCATTGACTGGCTCCAGC
Rfx3	mRfx3_F	GGGCTACGAACTAGGAGATTAGG
	mRfx3_R	CTTCTCCAAACTCTGGAAGTGCTCG
Rfx4	mRfx4_F	ACGGTGCTGCAGGCGTTGCCGGAC
	mRfx4_R	GTCTGCACTGTGGATCACCGTTCG
Rfx5	mRfx5_F	CCTCCTCCAAAGGCTCCGAGGTACC
	mRfx5_R	GGTGGTTGCGGATCCACCTATAGGC
Rfx7	mRfx7_F	AACTCTACCTCTACCTTCAACTGCC
	mRfx7_R	AATGGATGGTAGCCAAGATTGTCGC
Rfx8	mRfx8_F	TGCTCAGAACCAAGTCAACCCAGCG
	mRfx8_R	TTCTGAATGGAGACACAGGTGAGCC
Gli2	mGli2_F	GTGCACAGCAGCCCCACACTCTC
	mGli2_R	GGTAATAGTCTGAAGGGTTGGTGCCTGG
Gli3	mGli3_F	AACCCTATTCTACCCTCCAAA
	mGli3_R	GCTGATAGTGCTGGTATTGCT
Smo	mSmo_F	CGCTCTTCACACTGGCCACCTTTGTG
	mSmo_R	ATCTCATGGTGCCATCTGCTCGGC
Sufu	mSufu_F	CTCCAGGTTACCGCTATCGTC
	mSufu_R	CACTTGGTCCGTCTGTTCCTG
Gpr161	mGpr161_F	GACTAGCTCCATCCGGAGGGAATGG
	mGpr161_R	CATCAAACTCCACCGATGACCAACC
Ift27	mIft27_F	GACCCATTTCCAGAAGAACTACACC
	mIft27_R	GGGAGATGCCTGAAGTCTGAGACCG
Nqo1	mNqo1_F	GGTAGCGGCTCCATGTACTC
	mNqo1_R	CATCCTTCCAGGATCTGCAT
GLI3	hGLI3R_F	ATGGAGCCCCACTACCACCCTCC
	hGLI3R_R	CTCCTGCATGGGGCGCATCTGTAGG

Supplementary Figure S11. List of QPCR primers used in this study. All primers are specific for mouse cDNA amplification, except the last two, which are for human GLI3.

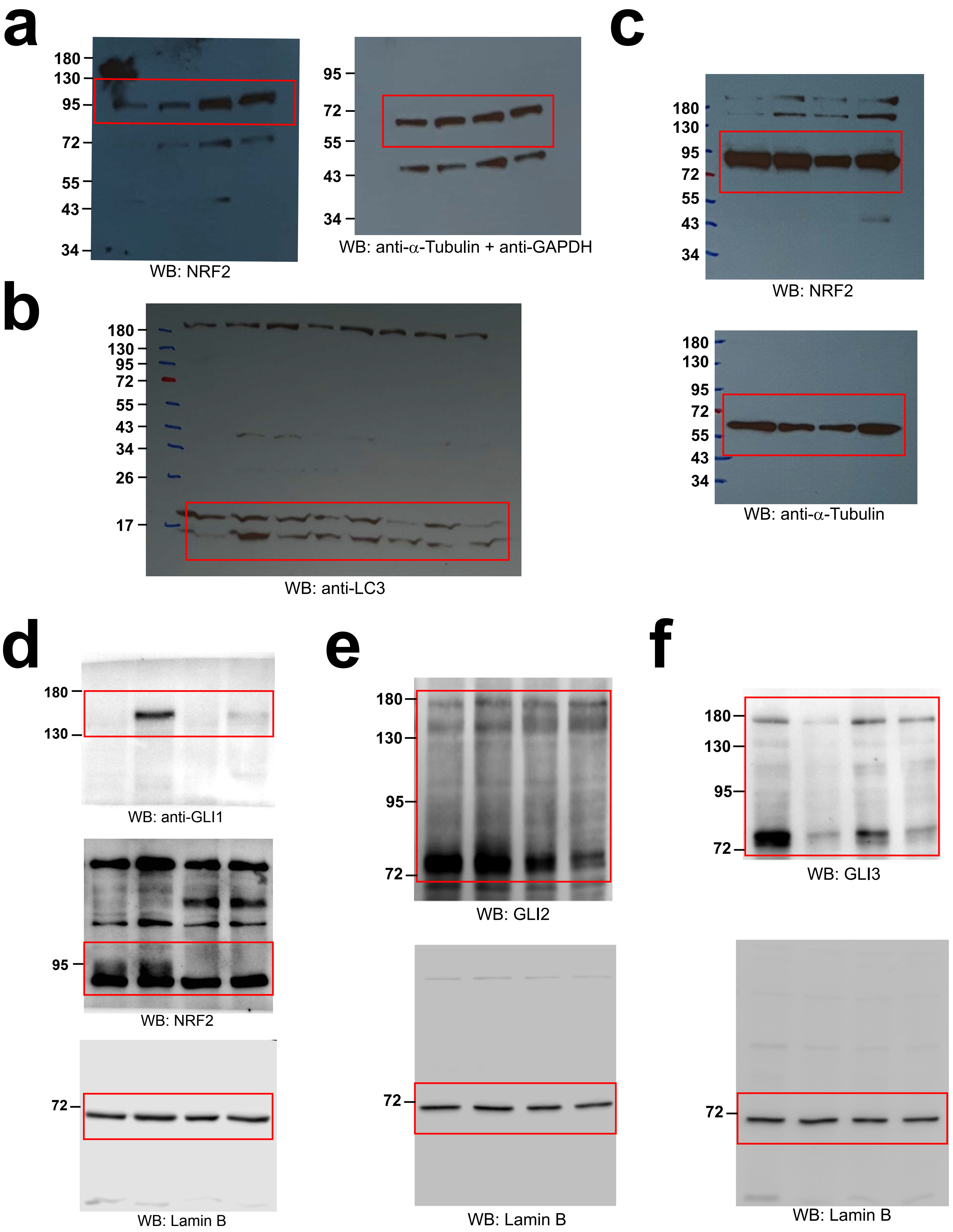

Supplementary Figure S12. Uncropped Western blots from main figures. (**a**) Blots from Figure 1d. Blot on the right was probed with mouse antibodies against both α-Tubulin (top band) and GAPDH (bottom band). (**b**) Blot from Figure 1g. (**c**) Blots from Figure 1h. (**d**) Blots from Figure 3b. (**e**) Blots from Figure 6e. (**f**) Blots from Figure 6f.
